# Plasma Extracellular Vesicles‐Derived Complement Proteins as Biomarkers of Sarcopenia Progression in Longitudinal Cohorts

**DOI:** 10.1002/jcsm.70350

**Published:** 2026-07-20

**Authors:** Ji Yeon Kim, Sung Hye Kong, Dohyun Han, Tae‐Hwan Gil, Hyeon Soo Kim, Miji Kim, Jin Hee Kim, Seung Shin Park, Seong‐Jun Park, Hak Chul Jhang, Chang Won Won, Ok Hee Jeon

**Affiliations:** ^1^ Department of Biomedical Sciences Korea University College of Medicine Seoul Republic of Korea; ^2^ Department of Internal Medicine Seoul National University Bundang Hospital Seongnam Republic of Korea; ^3^ Department of Internal Medicine Seoul National University College of Medicine Seoul Republic of Korea; ^4^ Department of Transdisciplinary Medicine Seoul National University Hospital Seoul Republic of Korea; ^5^ Department of Medicine Seoul National University College of Medicine Seoul Republic of Korea; ^6^ Department of Anatomy Korea University College of Medicine Seoul Republic of Korea; ^7^ Department of Biomedical Science and Technology, College of Medicine, East‐West Medical Research Institute Kyung Hee University Seoul Republic of Korea; ^8^ Department of Preventive Medicine and Public Health Ajou University School of Medicine Suwon Republic of Korea; ^9^ Department of Internal Medicine Seoul National University Hospital Seoul Republic of Korea; ^10^ PASS Center at Bertis Inc Gwacheon Gyeonggi‐do Republic of Korea; ^11^ Department of Family Medicine Kyung Hee University Medical Center Seoul Republic of Korea; ^12^ Elderly Frailty Research Center, Department of Family Medicine, College of Medicine Kyung Hee University Seoul Republic of Korea; ^13^ Department of Convergence Medicine Korea University College of Medicine Seoul Republic of Korea

**Keywords:** aging, biomarkers, complement proteins, extracellular vesicles, sarcopenia

## Abstract

**Background:**

Sarcopenia is characterized by progressive loss of muscle mass, strength and physical performance, yet current diagnostic tools are limited for practical and longitudinal monitoring. Extracellular vesicles (EVs) encapsulate various biomolecules, including proteins, nucleic acids, lipids and metabolites and have emerged as promising carriers of circulating biomarkers. In this study, we investigated longitudinal biomarkers of sarcopenia using plasma‐derived EVs.

**Methods:**

Plasma EVs were isolated by density gradient ultracentrifugation from participants in the community‐based Korean Frailty and Aging Cohort Study (KFACS, *n* = 90) and performed quantitative proteomic profiling of EVs derived from plasma. Candidate proteins were validated in an independent hospital‐based Osteoporosis Sarcopenia (OsteoSarc, *n* = 93) cohort at Seoul National University Bundang Hospital using MRM‐based LC–MS/MS. Multivariate models were adjusted for age, sex, body mass index (BMI) and relevant covariates: metabolic comorbidities (hypertension, myocardial infarction, peripheral artery disease, cerebrovascular disease, and diabetes mellitus) in the KFACS cohort and femoral neck bone mineral density in the OsteoSarc cohort.

**Results:**

In the KFACS cohort (mean age, 77.7 ± 4.1 years; range, 70.0–84.7; 50.0% women), EV‐associated complement proteins, particularly C2 and C4B, were strongly associated with declines in gait speed (β = −0.302, *p =* 0.006; β = −0.231, *p =* 0.028, respectively) and were elevated in individuals with worsened outcomes over 2 years (*p =* 0.002 and *p =* 0.034, respectively). Complement pathway enrichment analysis further supported associations with inflammatory and aging‐related signatures. Independent validation in the hospital‐based OsteoSarc cohort (mean age, 74.3 ± 12.0 years; range, 52.0–96.0; 88.2% women) confirmed the predictive value of C2 and C1R for gait speed and SPPB decline, with AUC values exceeding 0.7 for clinically relevant measures.

**Conclusion:**

Our findings demonstrate that several EV‐associated complement proteins, including C2, C4B and C1R, are strongly associated with longitudinal decline in gait speed, highlighting their potential as promising circulating biomarkers for sarcopenia progression.

## 1 Introduction

Sarcopenia is an age‐related muscle disease characterized by loss of skeletal muscle mass, strength, and physical performance [1]. Diagnosis relies on integrated assessments such as appendicular skeletal muscle mass/height^2^ (ASM/ht^2^), grip strength, gait speed, the 5‐times‐sit‐to‐stand test (5TST) and the Short Physical Performance Battery (SPPB) score, as recommended by international consensus groups such as the Asian Working Group for Sarcopenia (AWGS) [2]. However, these methods are time‐consuming and may be impractical for older adults with mobility limitations, highlighting the need for minimally invasive biomarkers for early detection and monitoring. Although several blood‐based biomarkers have been proposed [3], inconsistent results and cross‐sectional designs limit clinical use.

Extracellular vesicles (EVs) are lipid bilayer‐enclosed particles that carry bioactive molecules and circulate stably in plasma [4]. Their dynamic molecular cargo and systemic reach render them attractive for biomarker discovery in age‐related conditions, including sarcopenia [3, 4].

Our previous work using the OsteoSarcopenia (OsteoSarc) cohort demonstrated that the plasma and plasma‐derived EVs harbour biologically relevant molecules associated with sarcopenia [5, 6]. Specifically, plasma‐derived EVs‐enriched proteins, including myostatin, P3NP, and TNF‐α, were correlated with muscle function and physical performance. However, these studies were restricted to predefined candidates and did not explore broader proteomic patterns related to inflammation or tissue remodelling. Recent studies, including EXERNET‐Elder 3.0, identified EV‐associated PF4 and C1R as potential sarcopenia biomarkers [8], but these findings lacked longitudinal validation, and mechanistic insight remains limited.

To address this gap, we conducted a longitudinal proteomic analysis of plasma‐derived EVs from community‐ and hospital‐based cohorts followed over 2 years. This study aims to provide clinically actionable biomarkers for early risk assessment and intervention.

## 2 Materials and Methods

### 2.1 Study Design and Participants

**FIGURE 1:**
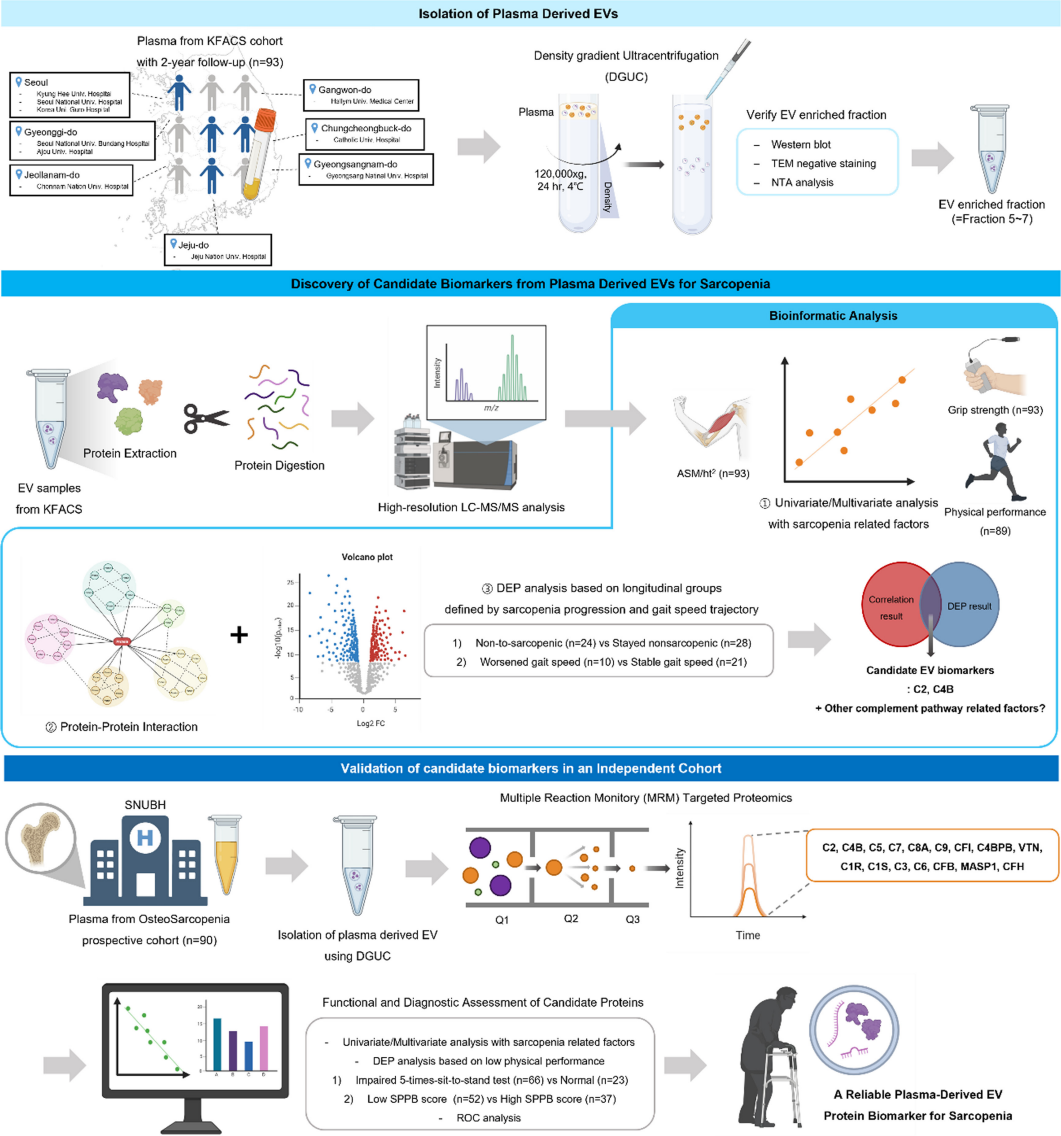
Schematic workflow of the study. KFACS, Korean Frailty and Aging Cohort Study; EV, extracellular vesicles; TEM, transmission electron microscopy; NTA, nanoparticle tracking analysis; SPPB, short physical performance battery; C2, Complement C2; C4B, Complement Component 4B; C5, Complement component 5; C7, Complement component 7; C8A, Complement Component 8 Alpha Chain; C9, Complement Component 9; CFI, Complement Factor I; C4BPB, Complement component 4 binding protein beta; VTN, Vitronectin; C1R, Complement C1r Subcomponent; C1S, Complement C1s Subcomponent; C3, Complement component 3; C6, Complement component 6; CFB, Complement Factor B; MASP1, Mannan‐Binding Lectin Serine Protease 1; CFH, Complement Factor H.

The study was designed as a longitudinal proteomic analysis with both discovery and validation phases (Figure 1). The discovery cohort was drawn from the Korean Frailty and Aging Cohort Study (KFACS), a nationwide, multicenter cohort that enrolled 3014 community‐dwelling older adults whose baseline assessments were performed between May 2016 and November 2017 [9]. Participants were categorized based on 2‐year sarcopenia status as ‘stayed nonsarcopenic’ (*n* = 31), ‘non‐to‐sarcopenic’ (*n* = 36) and ’Stayed sarcopenic’ (*n* = 23). Among participants with complete baseline and 2‐year follow‐up assessments, individuals who developed sarcopenia were first identified, and age‐ and sex‐matched controls and persistently sarcopenic participants were selected; the final analytic sample was further restricted to those with available plasma samples.

Independent validation was conducted in the OsteoSarc cohort at Seoul National University Bundang Hospital (SNUBH), Republic of Korea, initiated in 2021. By June 2024, 526 participants had been enrolled, with recruitment ongoing. Adults aged ≥ 50 years with a bone mineral density (BMD) *T*‐score ≤ − 2.5 or a history of osteoporotic fractures, and who were independently ambulatory, were eligible. Individuals without osteoporosis, or those with metabolic bone diseases or active malignancies, were excluded. For the present analysis, we included 93 individuals who had completed at least 2 years of follow‐up.

This study was approved by the Institutional Review Board of SNUBH (no. B‐2104‐678‐302), and written informed consent was obtained from all participants in accordance with the Declaration of Helsinki. Clinical characteristics of participants in both cohorts, including baseline and follow‐up assessments, are summarized in Table S1. Peripheral blood was collected in EDTA tubes, centrifuged to obtain plasma and stored at −80°C until analysis.

### 2.2 Demographic Characteristics

Information on participants' clinical characteristics, including age, sex and comorbidities such as diabetes mellitus (DM), hypertension (HTN), myocardial infarction (MI), peripheral artery disease (PAD) or cardiovascular disease (CVD), was collected through standardized health interviews and medical records. Height and body weight were measured to the nearest 0.1 cm and 0.1 kg while wearing light clothing, and BMI was calculated as weight (kg) divided by height (m^2^).

### 2.3 Assessment of Sarcopenia

ASM was assessed using dual‐energy X‐ray absorptiometry (Discovery W; Hologic, USA) and normalized to height squared (ASM/height^2^, m^2^). Grip strength was measured with a hydraulic dynamometer (Grip‐D 5401; Takei Scientific Instruments Co., Japan) with each hand tested three times, allowing a 60‐s rest between trials. The highest value was used for analysis. Physical performance was evaluated using the gait speed over a 4‐m distance; the faster of two trials was recorded. For the 5TST, participants were instructed to rise from a seated position five times in succession with arms folded across the chest, and the total time was recorded. SPPB scores, which comprise balance, 4‐m gait speed and 5TST, with higher scores reflect better physical performance. The balance test required maintaining side‐by‐side, semitandem and tandem stances for 10 s each.

In the discovery cohort, individuals who showed improvement over the 2‐year follow‐up, as well as those with persistent sarcopenia or those with consistently poor physical performance were excluded from further analyses.

### 2.4 Isolation EVs From Plasma

Plasma derived EVs were purified using differential gradient ultracentrifugation (DGUC), following the protocol described by Onodi et al. [10]. Optiprep solution (#07820, STEMCELL, Canada) was diluted to 50%, 30% and 10% concentrations using 0.25 M of sucrose buffer. Gradients were layered (1.17 mL each) into 3.7‐mL tubes (329561A, Himac, Germany), and 0.2 mL of plasma samples were gently overlaid atop the gradients. Ultracentrifugation was performed at 120000 × g for 24 h at 4°C using a P40ST rotor in the CP100WX ultracentrifuge (Himac, Germany). Ten fractions were collected sequentially from the top to the bottom, and each was stored at −80°C until use.

### 2.5 Nanoparticle Tracking Analysis (NTA)

Particle size distribution and concentration of plasma‐derived EVs were assessed using the NanoSight LM10 system (Malvern Panalytical, UK). For each sample, three 60‐s videos were recorded, with the camera level set to 15–16 to obtain the mean and mode vesicle size as well as particle concentration.

### 2.6 Transmission Electron Microscopy (TEM) With Negative Staining

The morphology and size of EVs were identified following a standard negative staining procedure [11]. Briefly, 30 μL of EV suspension was placed on Formvar–carbon grids (FCF300‐Cu, Electron Microscopy Sciences, USA) for 5 min. The grids were then immersed in 5% uranyl acetate for 5 min, rinsed twice with deionized water and blotted to remove excess liquid using filter paper. After air‐drying, the grids were visualized using the H‐7650 TEM system (Hitachi, Japan).

### 2.7 BCA Assay and Western Blot

The yield of plasma‐derived EVs was determined using the BCA Assay Kit (#23225, Thermo Scientific, USA). Absorbance was measured at 562 nm with a SpectraMax i3x multimode microplate reader (Molecular Devices, USA). About 964 μg of EV protein was obtained from 0.2 mL of plasma.

For western blotting, EV‐enriched fractions were prepared with 4 × LDS buffer (B0007, Thermo Scientific, USA). Samples were heated at 70°C for 10 min for the detection of ApoB, ApoE, CD9 and CD63, subjected to electrophoresis and transferred to PVDF membranes. Ponceau‐S staining (A40000279, Thermo Scientific, USA) was used to verify successful transfer. Membranes were blocked in 5% BSA in TBST (#28358, Thermo Scientific, USA) for 1.5 h and incubated with primary antibodies overnight at 4°C. Primary antibodies included anti‐CD9 (1:1000; GTX76185, GeneTex, USA), anti‐CD63 (1:1000; sc‐5275, Santa Cruz Biotechnology, USA), anti‐ApoB (1:1000; sc‐13 538, Santa Cruz Biotechnology, USA) and anti‐ApoE (1:1000; sc‐390 925, Santa Cruz Biotechnology, USA). Following TBST washing, membranes were exposed to HRP‐conjugated anti‐mouse (#7076) or anti‐rabbit (#7074) secondary antibodies (1:10000; Cell Signalling Technology, USA) for 1.5 h.

Protein bands were visualized using the SuperSignal West Femto substrate (#34094, Thermo Scientific, USA) and imaged with a ChemiDoc Touch Imaging System (Bio‐Rad, USA). Band intensities were quantified using Image Lab software (Bio‐Rad, USA) and normalized to the total signal per protein across fractions to assess relative enrichment.

### 2.8 Multiplex Surface Marker Analysis

Surface marker profiling of EVs was performed using the MACSPlex EV kit IO (human; #130‐122‐209, Miltenyi Biotec, Germany), following the manufacturer's instructions. The overnight incubation protocol was applied for capture antibody binding, and detection was carried out using CD9‐APC‐conjugated antibodies. Each assay used 20 μg of plasma‐derived EV and was analysed on the FACSCantoII (BD Biosciences, USA).

### 2.9 LC–MS/MS Analysis for Discovery Cohort

EV proteins were digested by automated filter‐aided sample preparation (aFASP) using a liquid‐handling robotic system (Agilent Technologies, USA). Peptides were analysed on an AB SCIEX TripleTOF mass spectrometer coupled to an Ekspert nanoLC 425 system (Eksigent, USA) in SWATH‐MS mode (400–1250 m/z). The resulting SWATH‐MS data were analysed using DIA‐NN (v1.8) in Human Swiss‐Prot database (January 2022), with a precursor‐level false discovery rate of 1%. Protein quantification was performed using the diann‐rpackage in R, followed by log2 transformation, filtering based on valid values, imputation and statistical analysis using Student's t‐test (*p* < 0.05) in Perseus (v2.0.9). Proteomics data are available via Table S2 and PRIDE (ProteomeXchange, PXD073288).

### 2.10 Peptide Quantification by Multiple Reaction Monitoring (MRM)‐Based LC–MS/MS

Proteins were extracted using Q700 sonicator (Qsonica, USA), reduced with DTT to 10 mM. Alkylation was performed with 25 mM of IAA for 30 min in the dark at room temperature. After adding 100 mM of ABC, trypsin (Promega, USA) was added to the enzyme at a protein ratio of 1:25 (w/w). Peptides were purified with SOLA HRP SPE 30 mg/2 mL 96‐well plates (#60509–001, Thermo Scientific, USA), dried and analysed using a TQ6500 + triple quadrupole MS (SCIEX, USA) coupled to an Eksigent Ekspert NanoLC 425 (SCIEX, USA) with a ZORBAX 300SB‐C18 column (#5064–8268, Agilent, USA). Relative peptide abundance was calculated using MultiQuant (v3.0.3, SCIEX, USA) based on the peak area ratio to iRT standards. Proteomics data are available in Table S3.

### 2.11 Pathway and Network Analyses

Canonical pathways were analysed with ingenuity pathway analysis (IPA; QIAGEN, Germany) with significance at *p* < 0.05 (Fisher's test) and activation inferred by *Z*‐scores ≥ 1. Protein–protein interactions and gene ontology biological process (GO‐BP) enrichment were analysed with STRING (version 12.0) [12], with confidence ≥ 0.4 and FDR ≤ 0.05.

### 2.12 Statistical Analysis

Associations between peptides and sarcopenia‐related factors were examined using univariate and multivariate linear regression. Discovery models were adjusted for age, sex, BMI, HTN, MI, PAD, CVD and DM; validation models for age, sex, BMI and femoral neck BMD. Participants with missing data due to poor physical condition were excluded: 34 from muscle mass analyses and 40 from muscle function and physical performance analyses. All variables were standardized (mean = 0, SD = 1), and analyses were performed in R (v4.4.2). Differentially expressed peptides and proteins were identified using Student's *t*‐test (*p* < 0.05) based on AWGS 2019‐defined sarcopenia‐related groupings [2]. ROC analyses were conducted using AUROC with 95% CIs, and optimal cutoffs were defined by the Youden index. Publicly available transcriptomic datasets (GSE145480 [13], GSE78702 [13], GSE146976 [14] and GSE28422 [15]) and raw proteomic data from Aparicio et al. [16] were independently analysed for the validation.

## 3 Results

### 3.1 Clinical Characteristics of Two Cohorts for EV‐Based Biomarkers of Sarcopenia Progression

Table 1 presents the baseline and follow‐up characteristics of participants in both the discovery (*n* = 90) and validation (*n* = 93) cohorts, each followed for 2 years. The discovery cohort was drawn from the KFACS [9], a nationwide, prospective cohort of community‐dwelling older adults, while the validation cohort was sourced from the OsteoSarc prospective cohort at SNUBH [5, 6].

**TABLE 1:** Baseline characteristics of the study population.

Variable	Discovery cohort (KFACS) *n* = 90			Validation cohort (OsteoSarc) *n* = 93		
	Baseline (mean ± SD)	Follow‐up (mean ± SD)	*p*	Baseline (mean ± SD)	Follow‐up (mean ± SD)	*p*
Gender, female, *n* (%)	45 (50.0)	45 (50.0)	1.00	82 (88.2)	82 (88.2)	1.00
Age, years	77.77 ± 4.14	79.77 ± 4.14	**<** **0.01**	74.31 ± 12.09	76.31 ± 12.09	**<** **0.01**
Body mass index, kg/m^2^	23.17 ± 2.63	23.02 ± 2.73	0.343	22.15 ± 4.19	21.97 ± 4.39	0.674
Diabetes mellitus, *n*a	25 (27.7)	20 (22.0)	0.511	9 (9.7)	9 (9.7)	1.00
Hypertension, *n* ^a^	56 (62.2)	50 (54.9)	0.430	32 (34.4)	32 (34.4)	1.00
Myocardial infarction, *n* (%)^a^	1 (1.1)	1 (1.1)	1.00	5 (5.4)	5 (5.4)	1.00
Peripheral artery disease, *n* (%)^a^	1 (1.1)	0 (0.0)	—	N/A	N/A	—
Cerebrovascular disease, *n* (%)^a^	4 (4.4)	3 (3.3)	1.00	N/A	N/A	—
SARC‐F, score	1.04 ± 1.47	1.07 ± 1.70	0.932	2.05 ± 2.11	N/A	—
Sarcopenia, *n* (%)^a^	23 (25.3)	59 (64.8)	**<** **0.01**	54 (60.6)	12 (33.3)	0.087
Appendicular skeletal muscle mass/heigh*t* ^2^, kg/m^2^ (*n*)	5.99 ± 1.00 (90)	5.57 ± 0.90 (90)	**<** **0.01**	4.93 ± 0.80 (93)	4.88 ± 0.84 (59)	0.742
Hand grip strength, kg (*n*)	24.45 ± 6.44 (90)	23.18 ± 6.83 (90)	**<** **0.01**	18.78 ± 5.94 (93)	19.09 ± 5.83 (53)	0.524
Gait speed, m/s (*n*)	0.99 ± 0.23 (90)	0.95 ± 0.25 (90)	**<** **0.01**	**0.79 ± 0.23 (89)**	**0.64 ± 0.16 (53)**	**<** **0.01**
Short physical performance battery, score (*n*)	10.75 ± 1.48 (87)	10.55 ± 1.41 (87)	0.230	**8.76 ± 2.45 (89)**	**7.77 ± 1.36 (53)**	**<** **0.01**
5‐times‐sit‐to‐stand test, s (*n*)	11.72 ± 4.10 (88)	12.16 ± 5.80 (90)	0.856	**19.55 ± 14.20 (89)**	**15.62 ± 13.94 (53)**	**0.003**

*Note:* All of ± data indicate mean ± standard deviation. The *p‐*values were calculated using the Wilcoxon test and the McNemar test. Statistically significant values (*p* < 0.05) are shown in bold.

Abbreviations: ADL, activities of daily living; BMD, bone mineral density; IADL, instrumental activities of daily living; KFACS, Korean Frailty and Aging Cohort Study; K‐MMSE, Korean mini mental state exam; P3NP, procollagen 3 N‐terminal peptide; SARC‐F, Strength, assistance with walking, rising from a chair, climbing stairs, and falls; SBNUH, Seoul National University Bundang Hospital.

^a^ McNemar test.

In the discovery cohort, the mean age at baseline was 77.7 ± 4.1 (range 70.0–84.7) years, with 45 participants (50.0%) being female. At baseline, 25.3% of participants met the diagnostic criteria for sarcopenia, which increased to 64.8% at follow‐up (*p* < 0.01). Significant declines were also observed in sarcopenia‐related factors: ASM/ht^2^ declined from 5.99 ± 1.00 kg/m^2^ to 5.57 ± 0.90 kg/m^2^ (*p* < 0.01), while grip strength declined from 24.45 ± 6.44 kg to 23.18 ± 6.83 kg, and gait speed declined from 0.99 ± 0.23 m/s at baseline to 0.95 ± 0.25 m/s (all *p* < 0.01). The SPPB score also dropped from 10.71 ± 1.48 to 10.55 ± 1.41 (*p* = 0.230).

In the validation cohort (mean age 74.3 ± 12.0 years, range 52.0–96.0; 88.2% female), 60.0% were sarcopenic at baseline. Over 2 years, gait speed declined from 0.79 ± 0.23 to 0.64 ± 0.16 m/s and SPPB from 8.76 ± 2.45 to 7.77 ± 1.36 (both *p* < 0.01). In contrast, performance on the 5TST improved, with the average completion time decreasing from 19.55 ± 14.20 s to 15.62 ± 13.94 s (*p* < 0.01).

### 3.2 Characterization of Plasma EVs and Their Alterations in Sarcopenia

Plasma EVs were isolated from the discovery cohort using DGUC, and all 10 gradient fractions (F1–F10) were systematically evaluated to identify EV‐enriched fractions with minimal lipoprotein contamination. Fractions 5–7 (F5–F7) were identified as EV‐enriched based on enrichment of canonical EV markers (CD9 and CD63) and marked depletion of lipoprotein markers (Apo B and Apo E) (Figure S1A). TEM confirmed typical cup‐shaped EV morphology [17] in these fractions (Figure S1B) and NTA revealed average modal sizes of 203 ± 29 nm (F5), 218 ± 59 nm (F6) and 226 ± 23 nm (F7) (Figure S1C), which is consistent with the size range of EVs defined in the MISEV2023 guidelines [18]. Particle concentrations were 14.0 × 10^8^, 16.3 × 10^8^ and 29.1 × 10^8^ particles/mL for F5–7, respectively, yielding a total of approximately 19.8 × 10^8^ particles/mL (Figure S1D). Protein contamination was minimal compared to other fractions, as verified by BCA assay and Ponceau S staining (Figure S1D,E). Therefore, pooled F5–F7 were designated for subsequent EV proteomic analyses.

To assess whether sarcopenia status alters EV characteristics, EVs from five nonsarcopenic controls and five sarcopenia participants were compared at baseline, with group classification based on the AWGS 2019 criteria [1]. NTA revealed no significant differences in average particle size (nonsarcopenic: 135.0 ± 18.0 nm vs. sarcopenia: 143.5 ± 24.8 nm, *p* = 0.556) or particle concentration (3.9 × 10^8^ vs. 5.3 × 10^8^ particles/mL, respectively, *p* = 0.369) (Figure S2A,B). EV surface marker profiling using MACSPlex assay revealed strong expression of canonical EV markers (CD9, CD63 and CD81) and platelet markers (CD41b, CD42a and CD62P) in both groups, consistent with the known predominant platelet‐derived EVs in circulation [19], regardless of sarcopenia status (Figure S2C). In addition, enrichment of CD81, CD146, and ROR1 was observed in sarcopenia, with trends toward higher CD105 and CD44 expression (*p* = 0.063 and *p* = 0.071, respectively). CD146 and CD105 are established endothelial markers, and ROR1 and CD44 are cancer cell‐associated EV markers (Figure S2D) [19]. Notably, EVs expressing these markers have been reported to induce smooth muscle cell senescence and muscle atrophy [20], suggesting their associations with vascular and tissue remodelling processes linked to sarcopenia progression.

### 3.3 Plasma EV Protein signatures Associated With Clinical Progression of Sarcopenia

To investigate the relationship between plasma‐derived EV proteins and clinical indicators of sarcopenia, we performed multivariate regression analyses. At baseline, lower muscle mass (ASM/ht^2^) was significantly associated with lower levels of EV proteins involved in lipid metabolism and inflammation, such as APOB, APOA, ADIPOQ and A2M. Additionally, ECM‐related proteins, including FBLN1 and LGALS3BP, showed inverse correlations with ASM/ht^2^, suggesting a link between structural remodelling and muscle loss. In contrast, 2‐year increases in ASM/ht^2^ were positively correlated with EV proteins involved in ECM stabilization and protease regulation, including THBS1, A2M and VMF (Table S4).

For grip strength, lower baseline values were associated with elevated levels of several components of the complement cascade (C8G, C8A, C6, C5, and MASP1) as well as oxidative stress regulators (CAT and CA1), suggesting that immune activation and redox imbalance may contribute to muscle strength decline. During follow‐up, improvements in grip strength were positively correlated with anti‐inflammatory and immune‐regulatory proteins, including SERPINA3, CFP and C1QC (Table S5).

For physical performance assessed by gait speed, THBS1 and CFHR1 were significantly correlated to baseline walking ability. Over the 2 years, improvements in gait speed were associated with the upregulation of proteins involved in coagulation (F2, F13A1), ECM organization (FBLN1) and complement components (C2, C1QA and C4B) (Table 2). Complement activation also showed associations with other physical performance‐based measures, including the 5TST and the SPPB score (Table S6). In particular, MASP1—a key protease involved in complement activation—levels were elevated in individuals with both poorer initial performance and in those who experienced greater physical performance decline during follow‐up.

**TABLE 2:** Unadjusted and adjusted associations between markers in plasma‐derived EV and gait speed.

Gait speed at baseline						Gait speed over a 2‐year period					
Univariate			Multivariate			Univariate			Multivariate		
Protein	β	*p*	Protein	β	*p*	Protein	β	*p*	Protein	β	*p*
			THBS1	−0.228	**0.018**	F2	−0.381	**0.000**	F2	−0.364	**0.001**
			CFHR1	0.219	**0.021**	QSOX1	−0.297	**0.004**	FBLN1	0.290	**0.006**
						F13A1	−0.287	**0.006**	C2	−0.302	**0.006**
						C1QA	0.282	**0.007**	F13A1	−0.289	**0.006**
						CP	−0.279	**0.008**	QSOX1	−0.295	**0.009**
						A2M	0.278	**0.008**	C1QA	0.272	**0.010**
						C2	−0.271	**0.010**	CP	−0.280	**0.010**
						ECM1	−0.264	**0.012**	ECM1	−0.273	**0.011**
						FBLN1	0.262	**0.013**	A2M	0.280	**0.012**
						MBL2	−0.235	**0.026**	MBL2	−0.247	**0.025**
						TTR	0.232	**0.028**	C4B	−0.231	**0.028**
						C4B	−0.227	**0.031**	SAA4	−0.240	**0.033**
						SELENOP	−0.224	**0.034**	SELENOP	−0.220	**0.038**
						CFHR5	−0.219	**0.038**	SERPINF1	−0.217	**0.042**
						GPX3	−0.215	**0.042**	COMP	−0.222	**0.045**
									CNDP1	−0.212	**0.049**

*Note:* β; standardized coefficient beta calculated by multivariable regression analysis adjusted for age, sex, BMI, HTN, MI, PAD, cerebro and DM. Statistically significant values (*p* < 0.05) are shown in bold.

Using these EV protein sets, defined as proteins significantly associated with baseline status or longitudinal changes in clinical sarcopenia indicators (ASM/ht^2^, grip strength, and gait speed), we next performed IPA to explore pathway‐level biological relevance, with results summarized in Tables S7–S12. Notably, the complement system was uniquely characterized by both statistical enrichment and a consistent directional *z*‐score in relation to sarcopenia progression. Particularly, improvements in physical performance over 2 years were associated with suppression of the complement system pathway, as reflected by decreased levels with suppression of key complement components, including C1QA, C2, C4B and MBL2 (Table S12).

### 3.4 Complement‐Associated EV Proteomic Signatures in Sarcopenia Progression and Aging

**FIGURE 2:**
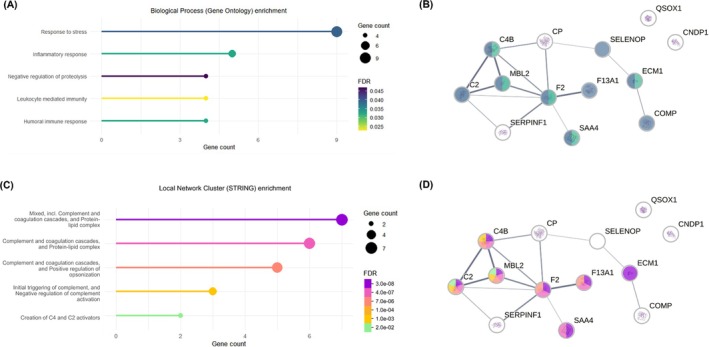
Functional enrichment and network analysis of EV proteins associated with longitudinal gait speed decline. (A) Gene ontology Biological Process (GO BP) enrichment analysis of EV proteins significantly downregulated in association with 2‐year gait speed decline. (B) Protein–protein interaction network of the downregulated proteins identified in (A). Proteins involved in the enriched GO BP terms ‘response to stress’ and ‘inflammatory response’ are marked in (B) using the same colour scheme as shown in the lollipop plot in (A). (C) STRING pathway enrichment analysis of the downregulated proteins from plasma‐derived EVs with longitudinal changes in gait speed. (D) Network analysis showing the role of downregulated EV proteins with longitudinal changes in gait speed in complement system. Line thickness indicates the strength of data support.

To investigate the biological processes underlying the decline in physical performance, we performed GO‐BP analysis on EV proteins associated with 2‐year gait speed deterioration. Notably, several significantly enriched GO terms were related to aging, such as ‘response to stress’ and ‘inflammatory response’ (Figure 2A) [21]. STRING network analysis further revealed significant enrichment in complement‐related terms (Figure 2C). Notably, many proteins were shared between the aging‐associated GO terms and the STRING‐derived complement network, including C2, C4B, MBL2, F2, F13A1, SAA4 and ECM1 (Figure 2B,D), suggesting a mechanistic link between complement activation and aging‐associated molecular changes linked to physical performance decline.

**FIGURE 3:**
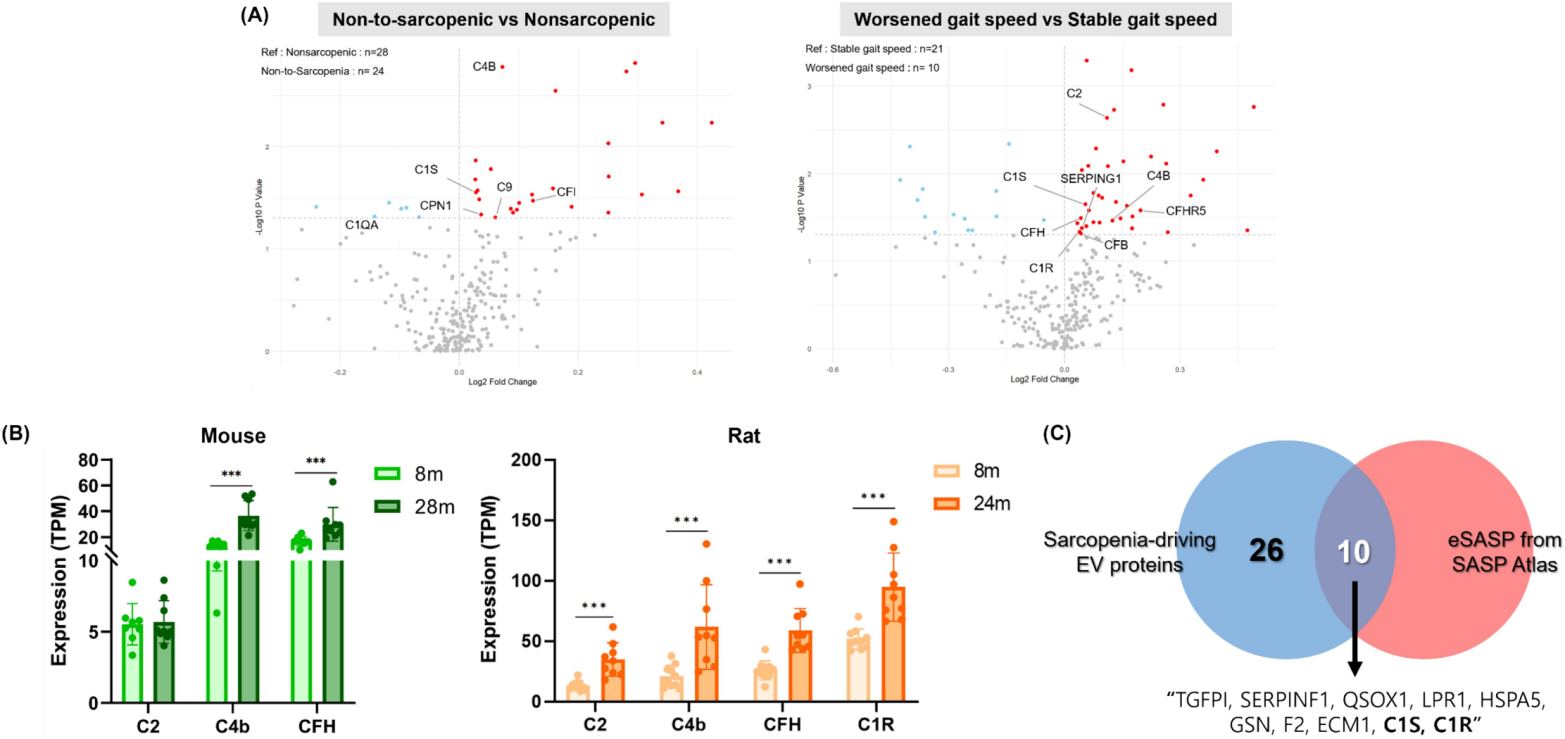
Complement‐related molecular signatures associated with sarcopenia progression and aging (A) Volcano plots showing differentially expressed plasma EV proteins based on normalized intensities between the non‐to‐sarcopenic versus stayed nonsarcopenic groups (left) and the worsened gait speed versus stable gait speed groups (right). Proteins with *p* > 0.05 are shown in grey. Among the significant proteins (*p* ≤ 0.05), those with positive fold change are shown in red, while those with negative fold change are shown in sky blue. The *p*‐values were calculated using the Mann–Whitney test. (B) Transcript‐level expression of complement‐related genes in aging gastrocnemius muscle from publicly available mouse and rat transcriptomic datasets (GSE145480) ^17^. Data are shown for young (8 months) and old (28 months for mice, 24 months for rat) groups. The *p‐*values were calculated by unpaired *t*‐test; *p* < 0.001 (***) (C) Venn diagram illustrating the overlap between EV proteins associated with sarcopenia progression and exosomal cargo SASP proteins (eSASP) annotated in SASP atlas ^19^. C2, Complement C2; C4B, Complement Component 4B Copy 2; C1R, Complement C1r Subcomponent; C1S, Complement C1s Subcomponent; CFH, Complement Factor H; CFB, Complement Factor B. CFI, Complement Factor I; C1QA, Complement C1q Subcomponent Subunit A; C9, Complement Component 9; MASP1, Mannan‐Binding Lectin Serine Protease 1; C8A, Complement Component 8 Alpha Chain; CPN1, Carboxypeptidase N Subunit 1; SERPING1, Serpin Family G Member 1.

To further explore molecular signatures of sarcopenia progression, we performed differential expression protein (DEP) analysis based on changes in sarcopenia status over the 2‐year follow‐up period (Table S13). Compared to the ‘stayed nonsarcopenic’ group (*n* = 28), the ‘non‐to‐sarcopenic’ group (*n* = 24) exhibited significantly elevated levels of complement pathway‐related proteins, including C4B, C1S, CPN1, C9, CFI and C1QA (Figure 3A). We also assessed proteomic differences related to gait speed decline. EV proteins from participants with ‘Worsened gait speed’ (*n* = 10) were compared to those with ‘Stable gait speed’ (*n* = 21). Several complement cascade‐associated proteins—C2, C1S, C4B, SERPING1, CFH, CFHR5, C1R and CFB—were significantly upregulated in the ‘worsened gait speed’ group (Figure 3A). These findings reinforce the consistent association between complement activation and decline in physical performance across distinct definitions of sarcopenia progression.

These proteins are also upregulated at the mRNA level in aging muscle tissue, as demonstrated by analysis of publicly available gastrocnemius muscle transcriptomic datasets from aging mouse and rat models [13]. In mice, C4B and CFH showed significant increases between 8 and 28 months, while in rats, C2, C4B, CFH and C1R were elevated with advancing age (Figure 3B). Finally, to explore potential overlap with senescence‐associated secretory phenotype (SASP) components, we compared our EV proteomics dataset with the exosome SASP (eSASP) atlas [22] (Figure 3C). Of the 36 EV proteins associated with sarcopenia progression or gait speed decline, 10 overlapped with known eSASP proteins, including C1S and C1R [22]. Collectively, these cross‐comparisons of the plasma EV proteome changes associated with sarcopenia progression and physical decline reflect complement‐associated molecular changes and overlap with senescence‐related factors.

### 3.5 Validation of EV Complement Proteins as Biomarkers for Sarcopenia‐Related Physical Decline

To validate the findings from the discovery cohort, we selected 16 complement‐related proteins for targeted quantification using MRM. This panel included C2 and C4B, which were consistently elevated in association with sarcopenia progression and impaired physical performance across all discovery analyses, along with 14 additional complement proteins: C5, C7, C8A, C9, CFI, C4BPB, VTN, C1R, C1S, C3, C6, CFB, MASP1 and CFH. In the OsteoSarc cohort, we assessed the correlation between MRM‐quantified protein levels and clinical measures of sarcopenia using multivariate regression models.

**FIGURE 4:**
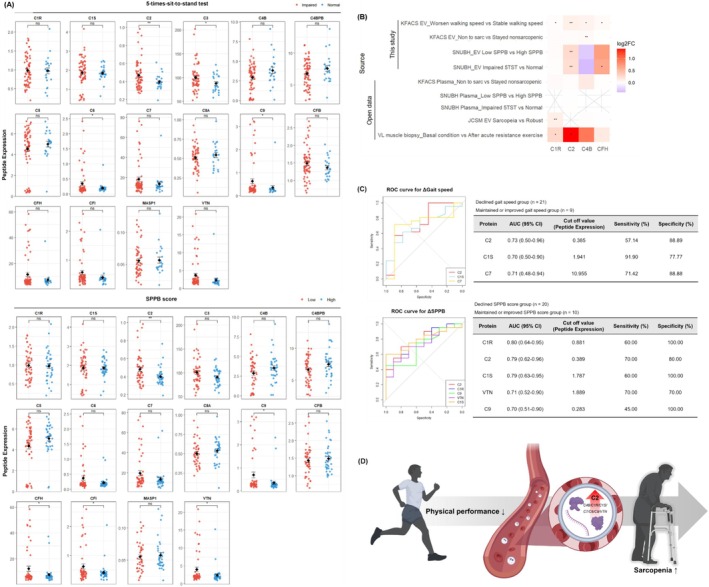
Validation of plasma‐derived EV markers reflecting sarcopenia‐related physical performance decline. (A) Dot plots of differentially expressed proteins based on peptide expression (endogenous peptide/iRT), comparing the Impaired 5‐times‐sit‐to‐stand test group (*n* = 66) vs. Normal (*n* = 23) (left) and the Low SPPB score group (*n* = 52) vs. High SPPB score group (*n* = 37) (right). The *p*‐values were determined by Mann–Whitney *U* test. (B) Comparative matrix summarizing the overlap of differentially expressed EV proteins identified in this study with those from previously published datasets related to sarcopenia, aging, or resistance exercise. Grey “X” indicates missing protein data in the reference dataset. (C) ROC curves of complement pathway‐related EV proteins in predicting physical performance decline, including Δgait speed (left) and ΔSPPB (right). (D) Schematic summary illustrating key EV proteomic alterations, particularly complement‐related proteins associated with decreased physical performance. *p* < 0.05 (*); *p* < 0.01 (**); ns, not significant.

Among the validated proteins, CFH was negatively associated with ASM/ht^2^. C2 was negatively correlated with grip strength and gait speed, while C1R also showed a significant inverse association with gait speed (Table 3). To further assess clinical relevance, we performed DEP analysis based on established clinical cutoffs for physical performance [1]. Individuals classified as ‘impaired’ on the 5TST (*n* = 66) had significantly higher EV levels of C2, C3, C6 and C9 than the ‘normal’ group (*n* = 23) (*p* < 0.05 for all). Similarly, in individuals with the ‘low’ SPPB scores (*n* = 52), EV levels of C2, C9, CFH, CFI and VTN were significantly elevated, while C5 and C4BPB were reduced compared to those in the ‘high’ score group (*n* = 37) (*p* < 0.05 for all) (Figure 4A).

**TABLE 3:** Unadjusted and adjusted associations between markers in plasma‐derived EV and sarcopenia‐related parameters in validation cohort.

	ASM/ht^2^				Grip strength				Gait speed			
	Univariate		Multivariate		Univariate		Multivariate		Univariate		Multivariate	
Protein	β	*p*	β	*p*	β	*p*	β	*p*	β	*p*	β	*p*
C1R	0.089	0.635	−0.169	0.140	−0.267	0.146	−0.298	0.141	**−** **0.381**	**0.035**	**−** **0.454**	**0.019**
CFB	**0.480**	**0.006**	−0.093	0.525	−0.123	0.509	−0.029	0.913	−0.014	0.940	−0.002	0.994
C3	**0.398**	**0.026**	−0.151	0.274	−0.099	0.597	−0.032	0.899	−0.166	0.372	−0.211	0.387
C5	0.307	0.093	−0.129	0.329	−0.106	0.570	−0.120	0.612	0.011	0.953	0.027	0.907
C9	−0.106	0.572	−0.062	0.567	−0.186	0.315	−0.192	0.314	0.083	0.659	0.044	0.818
VTN	**0.501**	**0.004**	0.121	0.371	−0.154	0.407	−0.181	0.454	0.023	0.901	0.007	0.976
CFI	**0.393**	**0.029**	−0.066	0.626	−0.106	0.569	−0.034	0.887	−0.251	0.173	−0.284	0.222
C2	0.343	0.059	−0.237	0.074	**−** **0.439**	**0.014**	**−** **0.543**	**0.018**	**−** **0.398**	**0.027**	**−** **0.537**	**0.018**
C8A	0.036	0.849	−0.190	0.107	−0.151	0.417	−0.159	0.459	0.184	0.323	0.207	0.325
CFH	0.033	0.862	**−** **0.339**	**0.003**	−0.230	0.213	−0.319	0.142	−0.179	0.335	−0.207	0.340
C1S	**0.367**	**0.042**	−0.018	0.883	−0.345	0.058	−0.278	0.202	−0.239	0.195	−0.254	0.240
C4B	0.218	0.240	−0.020	0.858	−0.046	0.806	−0.009	0.963	0.058	0.755	0.129	0.514
C7	**0.508**	**0.004**	0.092	0.481	−0.001	0.997	0.248	0.280	0.149	0.425	0.336	0.134
C6	−0.003	0.989	−0.013	0.912	−0.123	0.508	−0.195	0.350	0.130	0.485	0.051	0.806
C4BPB	0.014	0.938	−0.180	0.126	−0.106	0.570	−0.167	0.431	−0.156	0.402	−0.157	0.453
MASP1	−0.010	0.956	−0.062	0.650	−0.080	0.669	0.013	0.957	0.158	0.395	0.123	0.606

*Note: β*; standardized coefficient beta calculated by multivariable regression analysis adjusted for age, sex, BMI and Femoral neck(g/cm^2^). Statistically significant values (*p* < 0.05) are shown in bold.

To assess the robustness of our findings, we searched publicly available datasets for consistency in expression patterns of key proteins such as C1R, C2, C4B and CFH across various sarcopenia‐related and physical performance‐related cohorts (Figure 4B). Although plasma proteomic data from cohorts comparable to ours did not reach statistical significance (unpublished), EV samples from the EXERNET‐Elder 3.0 cohort (4 health centers, Zaragoza, Spain) [16] showed significant upregulation of C1R in individuals with sarcopenia compared to robust controls. In addition, transcriptomic data from human vastus lateralis muscle biopsies [15] showed that C1R and C2 were upregulated in response to acute resistance exercise.

To evaluate their potential as biomarkers for physical decline, we performed ROC curve analyses to predict future decline in SPPB scores, gait speed and 5TST performance (Figure 4C). For gait speed decline, C2, C1S and C7 achieved area under curve (AUC) values > 0.7, indicating moderate predictive power. For SPPB score decline, C2, C1R, C9, VTN, and C1S demonstrated slightly higher predictive performance. In contrast, no proteins achieved an AUC > 0.7 for predicting decline in the 5TST.

Across analyses, C2 consistently emerged as a key EV‐associated complement protein linked to sarcopenia progression, validated in both discovery and independent validation cohorts. Although proteins such as C4B, C1R, C1S, C7, C6, C9 and VTN showed cohort‐ or measure‐specific associations, their repeated links to physical impairment suggest broader involvement of complement‐related EV signatures as biologically informative markers sarcopenia progression‐associated biomarkers (Figure 4D). Together, these findings highlight plasma EV‐derived complement proteins, particularly C2, as promising biomarkers for early detection, risk stratification and potential therapeutic targeting in sarcopenia‐related physical decline.

## 4 Discussion

In this study, we analysed plasma‐derived EVs from two independent cohorts, a community‐based cohort (KFACS) and a clinical cohort (OsteoSarc), to investigate their potential as biomarkers of progression of sarcopenia. Through integrative proteomic profiling and clinical assessments, complement‐related EV proteins—particularly C2, C1R and C4B—were closely linked to longitudinal sarcopenia progression, especially declines in physical performance.

C2, a key component of the classical pathway of the complement system consistently emerged as a strong candidate biomarker, being associated with baseline physical performance and its longitudinal deterioration. In the discovery cohort, C2 was elevated in individuals who progressed to sarcopenia or experienced worsening physical performance during the 2‐year follow‐up compared with those who remained stable. In the validation cohort, C2 was significantly higher in participants with impaired physical performance. Importantly, C2 showed good predictive power for identifying participants at risk of decline in gait speed and SPPB score, with ROC values exceeding 0.7.

Other complement‐related EV proteins, including C4B and C1R, were also clinically informative. In the discovery cohort, both were significantly upregulated in participants with declining gait speed. In the validation cohort, C1R exhibited the strongest predictive power, achieving an AUC of 0.80 for discriminating participants with declining SPPB scores over 2 years. The reproducibility of complement‐associated EV signatures across two clinically distinct cohorts, community‐dwelling older adults and hospital‐based osteoporosis patients, supports the robustness of these associations despite differences in baseline sarcopenia prevalence and sex distribution.

These findings align with prior studies showing increased complement components such as C1R and C4B in aging‐ or sarcopenia‐related parameters, particularly physical performance [14, 15, 22]. This supports the biological plausibility of complement involvement in sarcopenia and age‐related muscle dysfunction. Complement activation may influence muscle aging through inflammatory mechanisms. Components of the classical complement pathway, including C1R and C2, initiate downstream cascades that generate inflammatory mediators such as C3a and C5a, which regulate macrophage recruitment and tissue remodelling [24]. While tightly regulated complement activity supports muscle repair, sustained low‐grade activation has been associated with chronic inflammation, impaired regeneration and fibrotic remodelling in aging muscle [25]. Complement proteins have also been identified as part of the SASP, suggesting that EV‐associated complement components may reflect systemic inflammaging processes [21, 25]. Although our findings support a biologically plausible link between complement activation and muscle aging, the present data do not establish direct causality, and the identified EV‐associated complement proteins should be interpreted as correlates of aging‐related inflammatory processes rather than confirmed mechanistic drivers.

Importantly, these findings should be interpreted within the context of the AWGS 2019 criteria, in which physical performance contributes to sarcopenia classification. Therefore, associations between identified EV proteins and gait speed represent functional‐domain associations that are clinically relevant to sarcopenia but should be distinguished from prediction of incident sarcopenia. Several candidate proteins identified in relation to gait speed also showed similar expression trends in comparisons between sarcopenic and non‐to‐sarcopenic participants, supporting their relevance to broader sarcopenia‐related functional decline. Additional analyses based on the AWGS 2025 criteria [27] in middle‐aged participants (50–64 years) from the validation cohort showed similar directional trends despite a lack of statistical significance, likely due to the limited sample size (Figure S3). These findings provide preliminary support for the relevance of the identified proteins across evolving diagnostic criteria that emphasize earlier recognition of individuals at risk for muscle health decline and warrant further investigation in larger cohorts.

The EV‐associated nature of these complement proteins appears critical when compared with plasma proteomic analyses. Although plasma and EV profiling were performed on different subsets of participants within the same overarching cohorts and heterogeneity in underlying clinical characteristics may have contributed to differences in statistical significance, complement proteins showed stronger and more consistent associations with sarcopenia‐related parameters in EV analyses than plasma proteomic analyses from the same cohorts [28]. These findings suggest that EV profiling may capture disease‐relevant signals that are less apparent in bulk plasma analyses.

From a biological perspective, circulating EVs in plasma originate from multiple tissues and cell types and can integrate signals from systemic and tissue‐specific biological processes associated with aging [29]. Thus, EV‐associated proteins may serve as composite indicators of age‐related physiological alterations rather than markers of a single tissue of origin. Several complement components identified in this study, including C1R, C2 and C4B, have been reported to be upregulated in aged skeletal muscle and in models of muscle dysfunction, supporting their relevance to muscle aging–related biological processes [14, 15]. Their detection within plasma‐derived EVs suggests that EVs may capture these aging‐associated molecular signatures in a stabilized and biologically meaningful form.

Building on our prior EV study using ELISA in a cross‐sectional cohort, the present study employed unbiased LC–MS proteomics in a longitudinal cohort. In the previous study, myostatin was significantly associated with baseline physical performance (*β* = −0.309, *p =* 0.014), whereas in the current study, EV‐associated proteins such as C2 (*β* = −0.302, *p =* 0.006) and C4B (*β* = −0.231, *p =* 0.028) were associated with longitudinal changes in physical performance [6]. In the independent validation cohort, baseline physical performance was significantly associated with C2 (β = −0.515, *p =* 0.008) and C1R (*β* = −0.578, *p =* 0.011). Compared with myostatin, complement‐associated EV proteins showed stronger associations with longitudinal functional decline in sarcopenia.

Several lines of evidence suggest that these EV complement proteins may reflect underlying aging‐related biological processes. C1R is listed in the eSASP in the SASP Atlas, implicating it in senescence‐related inflammation [22]. While C2 and C4B were not originally classified as eSASP factors, all three proteins have been reported to be upregulated in aged rodent skeletal muscle [13]. Minimal overlap, for example, HSPA5, was observed between grip strength‐associated eSASP proteins and the EV signatures identified here, suggesting that EV‐associated complement activation may reflect functional performance trajectories rather than static muscle strength alone. Collectively, these findings support the concept that EV‐associated complement proteins serve as biologically informative markers linking systemic aging and functional decline in muscle performance in sarcopenia [30, 31, 32]. Further studies are needed to determine whether these EV‐derived proteins contribute causally to muscle aging.

## 5 Limitations

While our study provides novel insights into plasma EV‐associated complement proteins as biomarkers of sarcopenia, several considerations should be noted. The validation cohort limited longitudinal follow‐up data, which constrained temporal analyses to baseline measurements. In addition, the modest sample size in the discovery cohort may have limited statistical power to detect small effect‐size proteins in the unbiased proteome‐wide analysis. The lack of proteins remaining significant after proteome‐wide FDR adjustment highlights the exploratory nature of the identified biomarkers and the need for further independent validation. Although C2, C1R and C4B emerged as candidate biomarkers associated with physical performance decline, the present observational and correlational design does not allow causal inference, and these proteins should be interpreted as biomarkers of aging‐related inflammation rather than direct mediators of sarcopenia. Whether these EV populations represent byproducts of vascular senescence, chronic inflammation or altered EV clearance in aging remains to be determined. Finally, while aging‐related associations were explored in the community‐based discovery cohort, further validation in larger and diverse populations is needed.

## 6 Conclusion

Our findings revealed that EV‐associated complement proteins (C2, C4B and C1R) are significantly associated with longitudinal declines in physical performance. These results highlight the potential of plasma‐derived EVs as a valuable source of circulating biomarkers for tracking sarcopenia progression. While further mechanistic validation is required, the consistent longitudinal associations observed across independent cohorts support their promise as progression‐linked biomarkers for monitoring functional decline in aging populations.

## Conflicts of Interest

The authors declare no conflicts of interest.

## Supporting information


**Figure S1:** Isolation and characterization of plasma‐derived of EVs from plasma (A) Western blot images of EV markers (CD9, CD63) and lipoproteins (ApoB, ApoE) across 10 density gradient ultracentrifugation (DGUC) fractions (F1–F10). Equal volumes of each fraction were analysed. The experiments were technically repeated three times. (B) Representative TEM images of plasma derived EVs. (C) Average particle size of each DGUC fraction measured by NTA (*n* = 3). (D) Particle concentration (bars) and total protein concentration (line) of each DGUC fraction (*n* = 3). Data are expressed as mean ± standard error of the mean. (E) Ponceau S staining of membranes after protein transfer, corresponding to immunoblots in (A).
**Figure S2:** Characterization of plasma‐derived EVs from nonsarcopenic controls and sarcopenia patients. (A) Average particle size and (B) concentration (particles/mL) measured by NTA of plasma‐derived EVs between healthy and sarcopenia groups. Data are expressed as mean ± standard deviation. (C) Surface marker profiling of plasma EVs using MACSPlex analysis. Dot plots display median fluorescence intensity (MFI) for selected markers, highlighting significant or trending differences between groups. All data are based on samples from each group (n = 5). (D) Heatmap displaying relative expression levels of 36 EV surface markers, with normalized MFI values, showing differences in marker distribution between groups. p < 0.05 (*); p < 0.01 (**); ns, not significant.
**Figure S3:** Expression patterns of candidate EV proteins according to the AWGS 2025 diagnostic criteria in middle‐aged participants of the validation cohort. (A) Schematic overview of the AWGS 2025 diagnostic algorithm [1]. (B) Expression levels of candidate EV proteins (C1R, C2 and C4B) in middle‐aged participants (50–64 years) from the validation cohort classified according to the AWGS 2025 criteria. Owing to the subgroup analysis restricted to middle‐aged participants, sample sizes for several comparisons were limited. Peptide expression levels were compared according to muscle mass status at baseline (low ASM/ht2, n = 22; normal, n = 3), muscle strength status at baseline (low grip strength, n = 13; normal, n = 12), sarcopenia status at baseline (sarcopenia, n = 13; normal, n = 12) and longitudinal changes in muscle mass, muscle strength and sarcopenia status. For longitudinal analyses, participants were classified according to changes in ASM/ht2 (stable, n = 3; worsen, n = 0), grip strength (stable, n = 4; worsen, n = 4) and sarcopenia status (stayed healthy, n = 4; nonsarcopenia to sarcopenia, n = 2). No participants in the middle‐aged subgroup were included in the worsen ASM/ht2 group because follow‐up ASM measurements were unavailable. p‐values were determined by the Mann–Whitney U test.

**Table S1:** Clinical characteristics according to sarcopenia progression groups at discovery and validation cohort.
**Table S4:** Unadjusted and adjusted associations between markers in plasma‐derived EV and muscle mass at baseline and over a 2‐year period.
**Table S5:** Unadjusted and adjusted associations between markers in plasma‐derived EV and muscle function at baseline and over a 2‐year period.
**Table S6:** Unadjusted and adjusted associations between proteins in plasma‐derived EV and other physical performance parameters.
**Table S13:** Differences in EVs‐related biomarker levels between stayed robust and non‐to‐sarcopenic groups over a 2‐year period.

**Table S2:** Processed LC?MS/MS proteomics dataset of plasma‐derived extracellular vesicles (EVs) from the KFACS cohort.
**Table S3:** Targeted MRM proteomics dataset of plasma‐derived extracellular vesicles (EVs) from the OsteoSarc cohort.

**TableS7:** Ingenuity pathway analysis (IPA) of proteins from plasma derived EVs with ASM/ht2 at baseline.
**Table S8:** Ingenuity pathway analysis (IPA) of proteins from plasma derived EVs with grip strength at baseline.
**Table S9:** Ingenuity pathway analysis (IPA) of proteins from plasma derived EVs with gait speed at baseline.
**Table S10:** Ingenuity pathway analysis (IPA) of proteins from plasma derived EVs with longitudinal changes in ASM.
**Table S11:** Ingenuity pathway analysis (IPA) of proteins from plasma derived EVs with longitudinal changes in grip strength.
**Table S12:** Ingenuity pathway analysis (IPA) of proteins from plasma derived EVs with longitudinal changes in gait speed.

## References

[1] A. J. Cruz‐Jentoft , G. Bahat , J. Bauer , et al., “Sarcopenia: Revised European Consensus on Definition and Diagnosis,” Age and Ageing 48 (2019): 601. 10.1093/ageing/afz046 PMC6593317 31081853

[2] L. K. Chen , J. Woo , P. Assantachai , et al., “Asian Working Group for Sarcopenia: 2019 Consensus Update on Sarcopenia Diagnosis and Treatment,” Journal of the American Medical Directors Association 21 (2019): 300–307.e2. 10.1016/j.jamda.2019.12.012 32033882

[3] J. Y. Reginster , C. Beaudart , N. Al‐Daghri , et al., “Update on the ESCEO Recommendation for the Conduct of Clinical Trials for Drugs Aiming at the Treatment of Sarcopenia in Older Adults,” Aging Clinical and Experimental Research 33 (2021): 3–17. 32737844 10.1007/s40520-020-01663-4 PMC7897619

[4] M. A. Kumar , S. K. Baba , H. Q. Sadida , et al., “Extracellular Vesicles as Tools And targets in Therapy for Diseases,” Signal Transduction and Targeted Therapy 9 (2024): 27. 38311623 10.1038/s41392-024-01735-1 PMC10838959

[5] X. Zhang , Y. Zhao , and W. Yan , “The Role of Extracellular Vesicles in Skeletal Muscle Wasting,” Journal of Cachexia, Sarcopenia and Muscle 14 (2023): 2462–2472. 37867162 10.1002/jcsm.13364 PMC10751420

[6] J. Y. Kim , T. H. Gil , H. G. Lee , et al., “Plasma Extracellular Vesicles Biomarkers Linked to Lower Muscle Mass, Function and Physical Performance in Sarcopenia,” Journal of Cachexia, Sarcopenia and Muscle 16 (2025): e13784. 40162588 10.1002/jcsm.13784 PMC11955922

[7] T. H. Gil , J. Y. Kim , J. W. Shin , et al., “Plasma Biomarkers for Monitoring Muscle Function and Physical Performance Decline in Older Adults: A 2‐Year Longitudinal Study,” Journal of Nutrition, Health & Aging 29 (2025): 100607. 10.1016/j.jnha.2025.100607 PMC12402374 40527083

[8] P. Aparicio , D. Navarrete‐Villanueva , A. Gómez‐Cabello , et al., “Proteomic Profiling of Human Plasma Extracellular Vesicles Identifies PF4 and C1R as Novel Biomarker in Sarcopenia,” Journal of Cachexia, Sarcopenia and Muscle 15 (2024): 1883–1897. 39009419 10.1002/jcsm.13539 PMC11446689

[9] C. W. Won , S. Lee , J. Kim , et al., “Korean Frailty and Aging Cohort Study (KFACS): Cohort Profile,” BMJ Open 10 (2020): e035573. 10.1136/bmjopen-2019-035573 PMC7204935 32327477

[10] Z. Onodi , C. Pelyhe , C. Terezia Nagy , et al., “Isolation of High‐Purity Extracellular Vesicles by the Combination of Iodixanol Density Gradient Ultracentrifugation and Bind‐Elute Chromatography From Blood Plasma,” Frontiers in Physiology 9 (2018): 1479. 30405435 10.3389/fphys.2018.01479 PMC6206048

[11] H. G. Lee , S. Roh , H. J. Kim , et al., “Nanoscale Biophysical Properties of Small Extracellular Vesicles From Senescent Cells Using Atomic Force Microscopy, Surface Potential Microscopy, and Raman Spectroscopy,” Nanoscale Horizons 7 (2022): 1488–1500. 36111604 10.1039/d2nh00220e

[12] D. Szklarczyk , R. Kirsch , M. Koutrouli , et al., “The String Database in 2023: Protein‐Protein Association Networks and Functional Enrichment Analyses for any Sequenced Genome of Interest,” Nucleic Acids Research 51 (2023): D638–D646. 36370105 10.1093/nar/gkac1000 PMC9825434

[13] A. Börsch , D. J. Ham , N. Mittal , et al., “Molecular and Phenotypic Analysis of Rodent Models Reveals Conserved and Species‐Specific Modulators of Human Sarcopenia,” Communications Biology 4 (2021): 194. 33580198 10.1038/s42003-021-01723-z PMC7881157

[14] A. Pannérec , M. Springer , E. Migliavacca , et al., “A Robust Neuromuscular System Protects Rat and Human Skeletal Muscle From Sarcopenia,” Aging (Albany NY) 8 (2016): 712–729. 27019136 10.18632/aging.100926 PMC4925824

[15] U. Raue , T. A. Trappe , S. T. Estrem , et al., “Transcriptome Signature of Resistance Exercise Adaptations: Mixed Muscle and Fiber Type Specific Profiles in Young and Old Adults,” Journal of Applied Physiology 112 (2012): 1625–1636. 22302958 10.1152/japplphysiol.00435.2011 PMC3365403

[16] P. Aparicio , D. Navarrete‐Villanueva , A. Gomez‐Cabello , et al., “Proteomic Profiling of Human Plasma Extracellular Vesicles Identifies PF4 and C1R as Novel Biomarker in Sarcopenia,” Journal of Cachexia, Sarcopenia and Muscle 15 (2024): 1883–1897. 39009419 10.1002/jcsm.13539 PMC11446689

[17] C. Thery , S. Amigorena , G. Raposo , and A. Clayton , “Isolation and Characterization of Exosomes From Cell Culture Supernatants and Biological Fluids,” Current Protocols in Cell Biology 30 (2006): 3.22.1–3.22.29. 10.1002/0471143030.cb0322s30 18228490

[18] J. A. Welsh , D. C. I. Goberdhan , L. O'Driscoll , et al., “Minimal Information for Studies of Extracellular Vesicles (MISEV2023): From Basic to Advanced Approaches,” Journal of Extracellular Vesicles 13 (2024): e12404. 38326288 10.1002/jev2.12404 PMC10850029

[19] U. Stok , E. Blokar , M. Lenassi , et al., “Characterization of Plasma‐Derived Small Extracellular Vesicles Indicates Ongoing Endothelial and Platelet Activation in Patients With Thrombotic Antiphospholipid Syndrome,” Cells 9, no. 5 (2020): 1211. 32414170 10.3390/cells9051211 PMC7290474

[20] M. J. Boyer , Y. Kimura , T. Akiyama , et al., “Endothelial Cell‐Derived Extracellular Vesicles Alter Vascular Smooth Muscle Cell Phenotype Through High‐Mobility Group Box Proteins,” Journal of Extracellular Vesicles 9 (2020): 1781427. 32944170 10.1080/20013078.2020.1781427 PMC7480479

[21] C. Lopez‐Otin , M. A. Blasco , L. Partridge , M. Serrano , and G. Kroemer , “Hallmarks of Aging: An Expanding Universe,” Cell 186 (2023): 243–278. 36599349 10.1016/j.cell.2022.11.001

[22] N. Basisty , A. Kale , O. H. Jeon , et al., “A Proteomic Atlas of Senescence‐Associated Secretomes for Aging Biomarker Development,” PLoS Biology 18 (2020): e3000599. 31945054 10.1371/journal.pbio.3000599 PMC6964821

[23] K. Wang , S. H. Smith , H. Iijima , et al., “Bioengineered 3D Skeletal Muscle Model Reveals Complement 4b as a Cell‐Autonomous Mechanism of Impaired Regeneration With Aging,” Advanced Materials 35 (2023): e2207443. 36650030 10.1002/adma.202207443 PMC13175247

[24] C. Zhang , C. Wang , Y. Li , et al., “Complement C3a Signaling Facilitates Skeletal Muscle Regeneration by Regulating Monocyte Function and Trafficking,” Nature Communications 8 (2017): 2078. 10.1038/s41467-017-01526-z PMC5727192 29233958

[25] R. Han , E. M. Frett , J. R. Levy , et al., “Genetic Ablation of Complement C3 Attenuates Muscle Pathology in Dysferlin‐Deficient Mice,” Journal of Clinical Investigation 120 (2010): 4366–4374. 21060153 10.1172/JCI42390 PMC2993587

[26] C. Franceschi , P. Garagnani , P. Parini , C. Giuliani , and A. Santoro , “Inflammaging: A New Immune‐Metabolic Viewpoint for Age‐Related Diseases,” Nature Reviews. Endocrinology 14 (2018): 576–590. 10.1038/s41574-018-0059-4 30046148

[27] L. K. Chen , F. Y. Hsiao , M. Akishita , et al., “A Focus Shift From Sarcopenia to Muscle Health in the Asian Working Group for Sarcopenia 2025 Consensus Update,” Nature Aging 5 (2025): 2164–2175. 41188603 10.1038/s43587-025-01004-y

[28] S. H. Kong , O. H. Jeon , J. Y. Kim , et al., “Distinct Proteomic Signatures Driving Progression of Sarcopenia: A Longitudinal Multicohort Study,” Journal of Cachexia, Sarcopenia and Muscle 17 (2026): e70240. 41782349 10.1002/jcsm.70240 PMC12961230

[29] Y. Li , X. He , Q. Li , et al., “EV‐Origin: Enumerating the Tissue‐Cellular Origin of Circulating Extracellular Vesicles Using exLR Profile,” Computational and Structural Biotechnology Journal 18 (2020): 2851–2859. 33133426 10.1016/j.csbj.2020.10.002 PMC7588739

[30] T. Tanaka , A. Biancotto , R. Moaddel , et al., “Plasma Proteomic Signature of Age in Healthy Humans,” Aging Cell 17 (2018): e12799. 29992704 10.1111/acel.12799 PMC6156492

[31] S. Sathyan , E. Ayers , T. Gao , et al., “Plasma Proteomic Profile of Age, Health Span, and All‐Cause Mortality in Older Adults,” Aging Cell 19 (2020): e13250. 33089916 10.1111/acel.13250 PMC7681045

[32] X. Liu , S. Pan , V. Xanthakis , et al., “Plasma Proteomic Signature of Decline in Gait Speed and Grip Strength,” Aging Cell 21 (2022): e13736. 36333824 10.1111/acel.13736 PMC9741503

[33] S. von Haehling , A. J. S. Coats , and S. D. Anker , “Ethical Guidelines for Publishing in the Journal of Cachexia, Sarcopenia and Muscle: Update 2023,” Journal of Cachexia, Sarcopenia and Muscle 14 (2023): 2981–2983. 38148513 10.1002/jcsm.13420 PMC10751405

