# Supplementary material for: Plasma Extracellular Vesicles‐Derived Complement Proteins as Biomarkers of Sarcopenia Progression in Longitudinal Cohorts
Source: J Cachexia Sarcopenia Muscle. 2026 Jul 20;17(4):e70350. doi: 10.1002/jcsm.70350 (PMC13385205; doi:10.1002/jcsm.70350)
Supplement: Supplementary file 1 — Figure S1: Isolation and characterization of plasma‐derived of EVs from plasma (A) Western blot images of EV markers (CD9, CD63) and lipoproteins (ApoB, ApoE) across 10 density gradient ultracentrifugation (DGUC) fractions (F1–F10). Equal volumes of each fraction were analysed. The experiments were technically repeated three times. (B) Representative TEM images of plasma derived EVs. (C) Average particle size of each DGUC fraction measured by NTA (n = 3). (D) Particle concentration (bars) and total protein concentration (line) of each DGUC fraction (n = 3). Data are expressed as mean ± standard error of the mean. (E) Ponceau S staining of membranes after protein transfer, corresponding to immunoblots in (A). Figure S2: Characterization of plasma‐derived EVs from nonsarcopenic controls and sarcopenia patients. (A) Average particle size and (B) concentration (particles/mL) measured by NTA of plasma‐derived EVs between healthy and sarcopenia groups. Data are expressed as mean ± standard deviation. (C) Surface marker profiling of plasma EVs using MACSPlex analysis. Dot plots display median fluorescence intensity (MFI) for selected markers, highlighting significant or trending differences between groups. All data are based on samples from each group (n = 5). (D) Heatmap displaying relative expression levels of 36 EV surface markers, with normalized MFI values, showing differences in marker distribution between groups. p < 0.05 (*); p < 0.01 (**); ns, not significant. Figure S3: Expression patterns of candidate EV proteins according to the AWGS 2025 diagnostic criteria in middle‐aged participants of the validation cohort. (A) Schematic overview of the AWGS 2025 diagnostic algorithm [1]. (B) Expression levels of candidate EV proteins (C1R, C2 and C4B) in middle‐aged participants (50–64 years) from the validation cohort classified according to the AWGS 2025 criteria. Owing to the subgroup analysis restricted to middle‐aged participants, sample sizes for several comparisons [file JCSM-17-e70350-s002.docx]

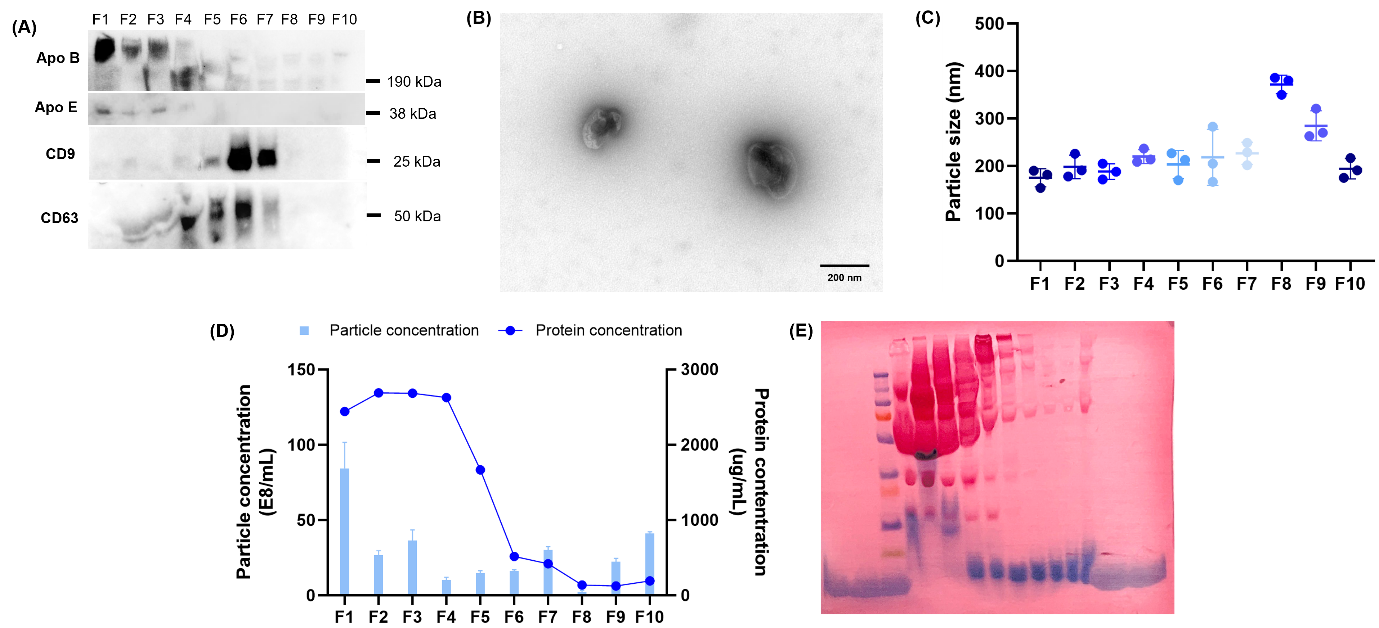


**Figure S1. Isolation and characterization of plasma-derived of EVs from plasma** (A) Western blot images of EV markers (CD9, CD63) and lipoproteins (ApoB, ApoE) across ten density gradient ultracentrifugation (DGUC) fractions (F1–F10). Equal volumes of each fraction were analyzed. The experiments were technically repeated three times. (B) Representative TEM images of plasma derived EVs. (C) Average particle size of each DGUC fraction measured by NTA (n = 3). (D) Particle concentration (bars) and total protein concentration (line) of each DGUC fraction (n = 3). Data are expressed as mean ± standard error of the mean. (E) Ponceau S staining of membranes after protein transfer, corresponding to immunoblots in (A).


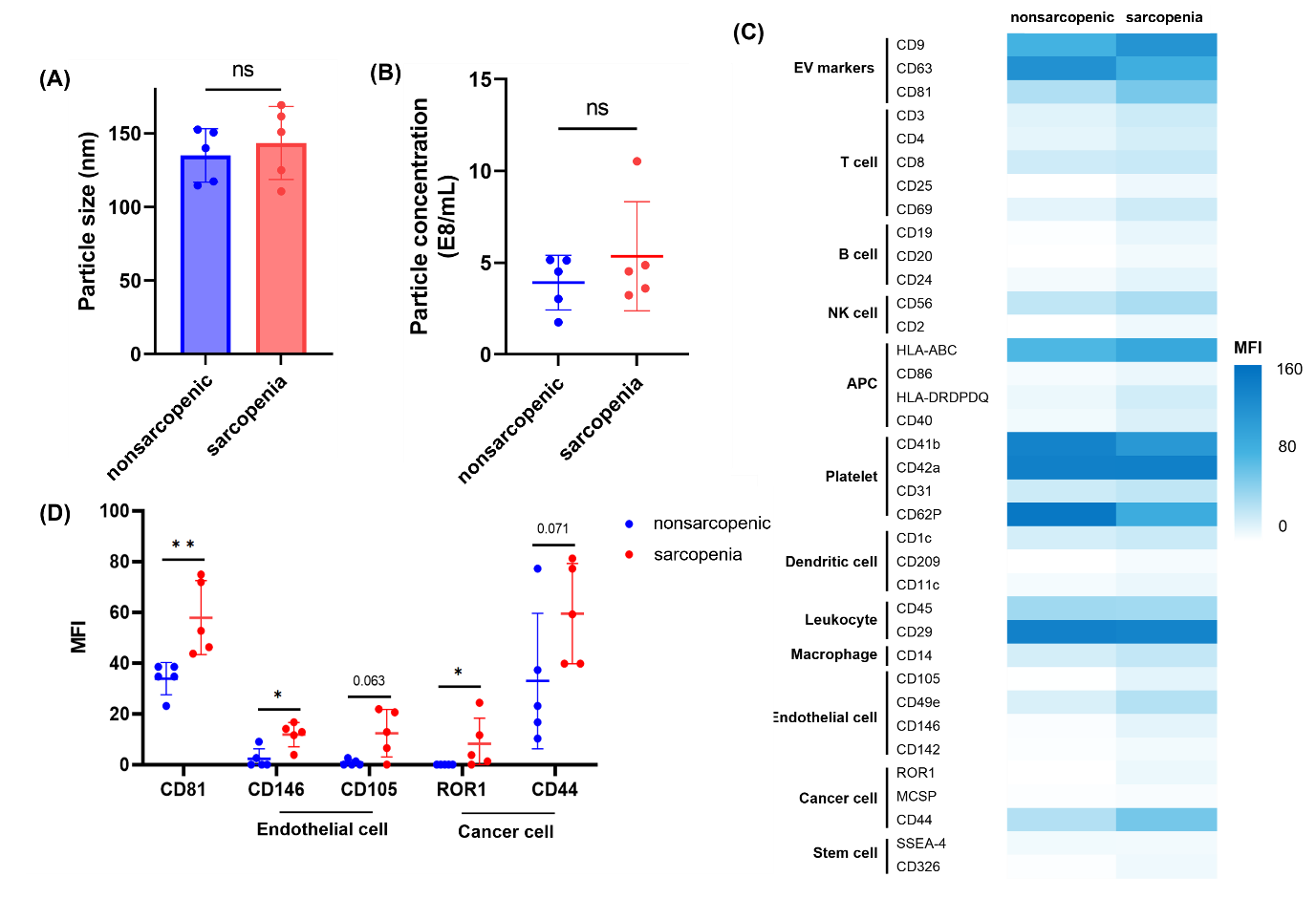


**Figure S2. Characterization of plasma-derived EVs from nonsarcopenic controls and sarcopenia patients.** (A) Average particle size and (B) concentration (particles/mL) measured by NTA of plasma-derived EVs between healthy and sarcopenia groups. Data are expressed as mean ± standard deviation. (C) Surface marker profiling of plasma EVs using MACSPlex analysis. Dot plots display median fluorescence intensity (MFI) for selected markers, highlighting significant or trending differences between groups. All data are based on samples from each group (n = 5). (D) Heatmap displaying relative expression levels of 36 EV surface markers, with normalized MFI values, showing differences in marker distribution between groups. *p* < 0.05 (*); p < 0.01 (**); ns, not significan


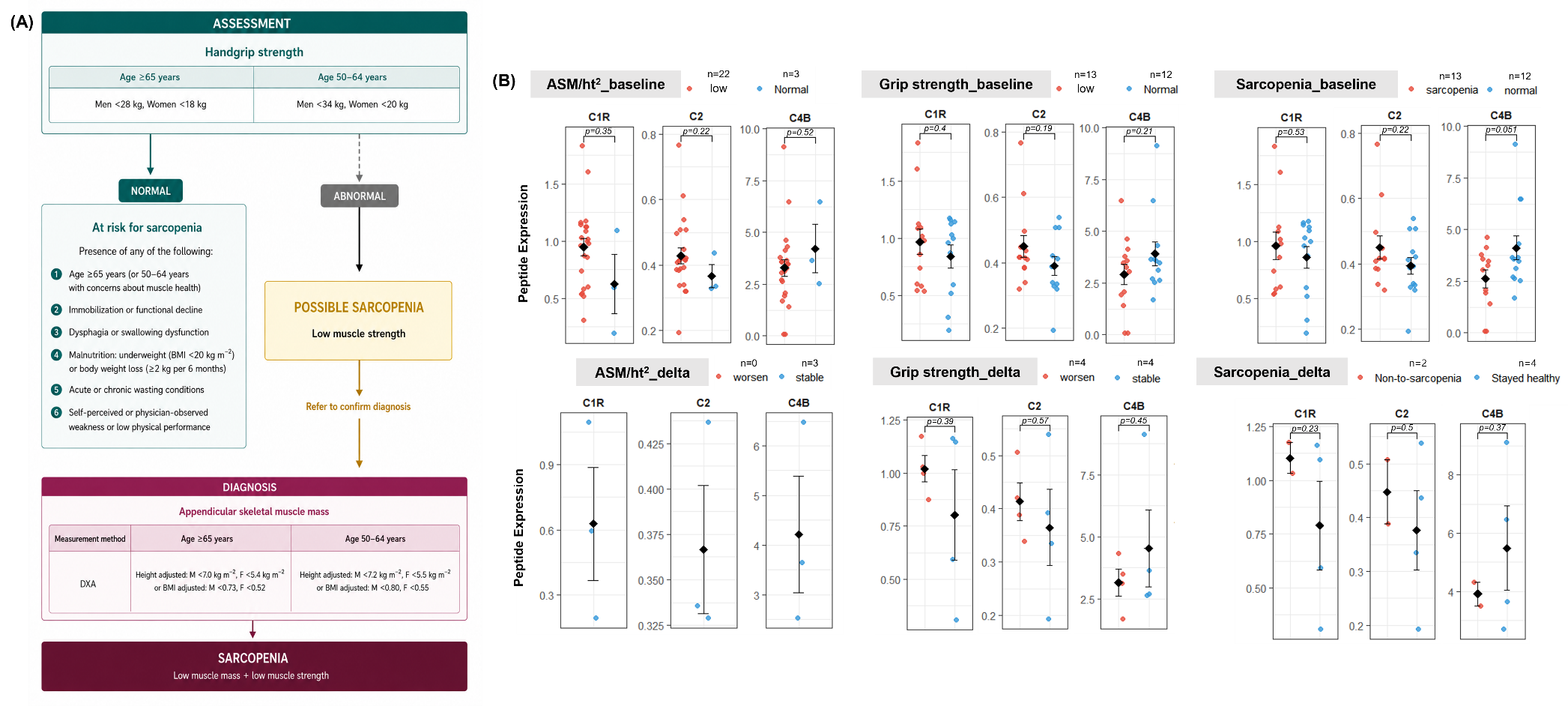


**Figure S3. Expression patterns of candidate EV proteins according to the AWGS 2025 diagnostic criteria in middle-aged participants of the validation cohort.** (A) Schematic overview of the AWGS 2025 diagnostic algorithm [28]. (B) Expression levels of candidate EV proteins (C1R, C2, and C4B) in middle-aged participants (50–64 years) from the validation cohort classified according to the AWGS 2025 criteria. Owing to the subgroup analysis restricted to middle-aged participants, sample sizes for several comparisons were limited. Peptide expression levels were compared according to muscle mass status at baseline (low ASM/ht², *n* = 22; normal, *n* = 3), muscle strength status at baseline (low grip strength, *n* = 13; normal, *n* = 12), sarcopenia status at baseline (sarcopenia, *n* = 13; normal, *n* = 12), and longitudinal changes in muscle mass, muscle strength, and sarcopenia status. For longitudinal analyses, participants were classified according to changes in ASM/ht² (stable, *n* = 3; worsen, *n* = 0), grip strength (stable, *n* = 4; worsen, *n* = 4), and sarcopenia status (stayed healthy, *n* = 4; non-sarcopenia to sarcopenia, *n* = 2). No participants in the middle-aged subgroup were included in the worsen ASM/ht² group because follow-up ASM measurements were unavailable. *p* values were determined by the Mann–Whitney U test.
