# Supplementary material for: Plasma Extracellular Vesicles‐Derived Complement Proteins as Biomarkers of Sarcopenia Progression in Longitudinal Cohorts
Source: J Cachexia Sarcopenia Muscle. 2026 Jul 20;17(4):e70350. doi: 10.1002/jcsm.70350 (PMC13385205; doi:10.1002/jcsm.70350)
Supplement: Supplementary file 2 — Table S1: Clinical characteristics according to sarcopenia progression groups at discovery and validation cohort. Table S4: Unadjusted and adjusted associations between markers in plasma‐derived EV and muscle mass at baseline and over a 2‐year period. Table S5: Unadjusted and adjusted associations between markers in plasma‐derived EV and muscle function at baseline and over a 2‐year period. Table S6: Unadjusted and adjusted associations between proteins in plasma‐derived EV and other physical performance parameters. Table S13: Differences in EVs‐related biomarker levels between stayed robust and non‐to‐sarcopenic groups over a 2‐year period. [file JCSM-17-e70350-s001.docx]

**Supplementary table 1. Clinical characteristics according to sarcopenia progression groups at discovery and validation cohort**

| **Discovery cohort (KFACS) n=90** | | | | | | | | |
| --- | --- | --- | --- | --- | --- | --- | --- | --- |
| **Variable** | **Baseline** | | | | **Follow up** | | | |
|  | **Stayed nonsarcopenic**  **(n=31)** | **Non-to-sarcopenic (n=36)** | **Stayed sarcopenic (n=23)** | ***p-value*** | **Stayed nonsarcopenic**  **(n=31)** | **Non-to-sarcopenic (n=36)** | **Stayed sarcopenic (n=23)** | ***p-value*** |
| **Gender, female n (%)** | 15 (16.7) | 22 (24.4) | 8 (8.9) | - | 15 (16.7) | 22 (24.4) | 8 (8.9) | - |
| **Age, years** | 76.62 ± 4.48 | 77.18 ± 3.74 | 80.24 ± 3.33 | **0.004** | 78.54 ± 4.49 | 79.09 ± 3.74 | 82.15 ± 3.33 | **0.004** |
| **Body mass index, kg/m^2^** | 22.92 ± 1.93 | 23.17 ± 2.72 | 23.49 ± 3.29 | 0.919 | 22.90 ± 2.15 | 23.18 ± 2.65 | 22.94 ± 3.53 | 0.898 |
| **Diabetes mellitus, n (%)** | 9 (10.0) | 9 (10.0) | 7 (7.8) | - | 7 (7.8) | 8 (8.9) | 5 (5.6) | - |
| **Hypertension, n (%)** | 21 (23.3) | 24 (26.7) | 11 (12.2) | - | 15 (16.7) | 23 (25.6) | 12 (13.3) | - |
| **Myocardial infarction, n (%)** | 1 (1.1) | 0 (0.0) | 0 (0.0) | - | 0 (0.0) | 0 (0.0) | 1 (1.1) | - |
| **Peripheral artery disease, n (%)** | 1 (1.1) | 0 (0.0) | 0 (0.0) | - | 0 (0.0) | 0 (0.0) | 0 (0.0) | - |
| **Cerebrovascular disease, n (%)** | 1 (1.1) | 3 (3.3) | 0 (0.0) | - | 1 (1.1) | 1 (1.1) | 1 (1.1) | - |
| **SARC-F, score** | 0.54 ± 0.76 | 1.55 ± 1.99 | 0.91 ± 0.94 | 0.077 | 0.41 ± 0.80 | 1.47 ± 2.13 | 1.34 ± 1.64 | **0.031** |
| **Appendicular skeletal muscle mass/ht^2^, kg/m^2^** | 6.30 ± 1.18 | 5.94 ± 0.93 | 5.65 ± 0.69 | 0.114 | 6.00 ± 0.98 | 5.37 ± 0.82 | 5.33 ± 0.72 | **0.014** |
| **Grip strength, kg** | 28.82 ± 6.16 | 22.74 ± 5.86 | 21.23 ± 4.31 | **<0.001** | 29.14 ± 6.42 | 19.35 ± 4.59 | 21.12 ± 4.55 | **<0.001** |
| **Gait speed, m/s** | 1.14 ± 0.25 | 1.01 ± 0.19 | 1.00 ± 0.15 | **0.034** | 1.23 ± 0.25 | 1.06 ± 0.23 | 1.01 ± 0.17 | **0.002** |
| **Short physical performance battery, score (n)** | 10.97 ± 1.35 (30) | 10.58 ± 1.77 (36) | 10.71 ± 1.10 (21) | 0.512 | 10.68 ± 1.37 (31) | 10.53 ± 1.50 (34) | 10.41 ± 1.36 (22) | 0.953 |
| **5-times-sit-to-stand test, sec (n)** | 10.88 ± 2.93 (30) | 12.20 ± 5.51 (36) | 12.05 ± 2.38 (22) | 0.216 | 10.81 ± 4.35 (31) | 12.62 ± 6.51 (36) | 13.69 ± 6.34 (23) | **0.035** |
| **Validation cohort (OsteoSarc) n=29** | | | | | | | | |
| **Variable** | **Baseline** | | | | **Follow up** | | | |
|  | **Stayed nonsarcopenic**  **(n=17)** | **Non-to-sarcopenic (n=1)** | **Stayed sarcopenic (n=11)** | ***p-value*** | **Stayed nonsarcopenic**  **(n=17)** | **Non-to-sarcopenic (n=1)** | **Stayed sarcopenic (n=11)** | ***p-value*** |
| **Gender, female n (%)** | 17 (58.6) | 1 (3.4) | 6 (20.7) | - | 17 (58.6) | 1 (3.4) | 6 (20.7) | - |
| **Age, years** | **74.05** ± 16.18 | **74.00** | **79.09** ± 8.67 | - | **76.05** ± 16.18 | **76.00** | **81.09** ± 8.67 | - |
| **Body mass index, kg/m^2^** | **22.45** ± 6.30 | **25.25** | **21.48** ± 3.07 | - | **22.30** ± 6.47 | **25.11** | **20.99** ± 3.41 | - |
| **Diabetes mellitus, n (%)** | **5 (17.2)** | **0 (0.0)** | **0 (0.0)** | - | **5 (17.2)** | **0 (0.0)** | **0 (0.0)** | - |
| **Hypertension, n (%)** | **7 (24.1)** | **1 (3.4)** | **1 (3.4)** | - | **7 (24.1)** | **1 (3.4)** | **1 (3.4)** | - |
| **Myocardial infarction, n (%)** | **0 (0.0)** | **0 (0.0)** | **0 (0.0)** | - | **0 (0.0)** | **0 (0.0)** | **0 (0.0)** | - |
| **SARC-F, score** | **1.23** ± 1.25 | **0.00** | **2.27** ± 1.90 | - | **-** | **-** | **-** | - |
| **Appendicular skeletal muscle mass/ht^2^, kg/m^2^** | **5.26** ± 1.12 | **5.35** | **4.91** ± 0.76 | - | **5.18** ± 1.10 | **5.21** | **4.75** ± 0.58 | - |
| **Grip strength, kg** | **20.68** ± 4.51 | **22.60** | **16.50** ± 5.59 | - | **20.88** ± 5.30 | **6.50** | **16.15** ± 4.33 | - |
| **Gait speed, m/s** | **0.84** ± 0.16 | **0.66** | **0.77** ± 0.31 | - | **0.72** ± 0.10 | **0.57** | **0.65** ± 0.15 (10) | - |
| **Short physical performance battery, score (n)** | **10** ± 1.73 | **11.00** | **8.27** ± 3.06 | - | **8.00** ± 1.00 | **11.00** | **8.00** ± 0.94 (10) | - |
| **5-times-sit-to-stand test, sec (n)** | **15.94** ± 12.61 | **10.00** | **21.72** ± 15.88 | - | **10.35** ± 3.80 | **19.00** | **11.23** ± 3.71 (10) | - |

KFACS, Korean Frailty and Aging Cohort Study; SNUBH, Seoul National University Bundang Hospital; SARC-F, Strength, assistance with walking, rising from a chair, climbing stairs, and falls. *P-values* were calculated using the Kruskal–Wallis test. Each ± data indicates the mean ± standard deviation. Samples (n=64) without follow-up data on ASM/ht^2^ or hand grip strength were excluded from the analysis.

**Supplementary table 4. Unadjusted and adjusted associations between markers in plasma-derived EV and muscle mass at baseline and over a 2-year period**

| **ASM/ht^2^ at baseline** | | | | | | **ASM/ht^2^ over a 2-year period** | | | | | |
| --- | --- | --- | --- | --- | --- | --- | --- | --- | --- | --- | --- |
| **Univariate** | | | **Multivariate** | | | **Univariate** | | | **Multivariate** | | |
| **Protein** | **ꞵ** | ***p-*value** | **Protein** | **ꞵ** | ***p-*value** | **Protein** | **ꞵ** | ***p-*value** | **Protein** | **ꞵ** | ***p-*value** |
| **F5** | -0.227 | **0.031** | **APOB** | -0.180 | **0.036** | **A2M** | 0.331 | **0.001** | **THBS1** | 0.222 | **0.045** |
| **PZP** | -0.254 | **0.016** | **APOA4** | -0.166 | **0.045** | **VWF** | 0.209 | **0.049** | **A2M** | 0.404 | **0.000** |
| **FBLN1** | -0.231 | **0.028** | **FBLN1** | -0.181 | **0.023** |  |  |  | **VWF** | 0.229 | **0.043** |
| **ADIPOQ** | -0.316 | **0.002** | **ADIPOQ** | -0.219 | **0.008** |  |  |  |  |  |  |
| **TTN** | 0.246 | **0.020** | **A2M** | -0.272 | **0.001** |  |  |  |  |  |  |
| **APOA2** | 0.226 | **0.032** | **LGALS3BP** | -0.201 | **0.013** |  |  |  |  |  |  |
| **JCHAIN** | -0.230 | **0.029** |  |  |  |  |  |  |  |  |  |

ꞵ; standardized coefficient beta calculated by multivariable regression analysis adjusted for age, sex, BMI, HTN, MI, PAD, cerebro, and DM.

**Supplementary table 5. Unadjusted and adjusted associations between markers in plasma-derived EV and muscle function at baseline and over a 2-year period**

| **Grip strength at baseline** | | | | | | **Grip strength over a 2-year period** | | | | | |
| --- | --- | --- | --- | --- | --- | --- | --- | --- | --- | --- | --- |
| **Univariate** | | | **Multivariate** | | | **Univariate** | | | **Multivariate** | | |
| **Protein** | **ꞵ** | ***p-*value** | **Protein** | **ꞵ** | ***p-*value** | **Protein** | **ꞵ** | ***p-*value** | **Protein** | **ꞵ** | ***p-*value** |
| **CAT** | 0.272 | **0.009** | **PRG4** | -0.181 | **0.031** | **PRPF19** | 0.245 | **0.020** | **PRPF19** | 0.237 | **0.029** |
| **PRG4** | -0.280 | **0.008** | **AFM** | 0.164 | **0.037** | **CFP** | 0.268 | **0.011** | **CFP** | 0.308 | **0.007** |
| **C6** | -0.250 | **0.017** | **C8B** | -0.152 | **0.048** | **APOC1** | -0.226 | **0.032** | **TGFBI** | -0.247 | **0.032** |
| **PZP** | -0.302 | **0.004** | **MMRN1** | -0.155 | **0.045** | **BNC2** | 0.222 | **0.036** | **APOC1** | -0.219 | **0.048** |
| **HBD** | 0.228 | **0.031** | **MASP1** | -0.167 | **0.034** | **PMEL** | -0.311 | **0.003** | **BNC2** | 0.234 | **0.035** |
| **ADIPOQ** | -0.222 | **0.036** | **SERPINA10** | -0.175 | **0.026** | **SERPINA3** | -0.208 | **0.049** | **PMEL** | -0.320 | **0.003** |
| **CA1** | 0.222 | **0.036** | **PRSS3** | -0.180 | **0.037** |  |  |  | **C1QC** | 0.235 | **0.038** |
| **SERPINA10** | -0.221 | **0.037** | **C5** | -0.172 | **0.032** |  |  |  | **AMBP** | -0.227 | **0.041** |
| **PRSS3** | -0.216 | **0.041** | **HBB** | 0.164 | **0.036** |  |  |  |  |  |  |
| **HBB** | 0.254 | **0.016** | **C8G** | -0.208 | **0.007** |  |  |  |  |  |  |
| **JCHAIN** | -0.285 | **0.006** | **C8A** | -0.183 | **0.017** |  |  |  |  |  |  |

ꞵ; standardized coefficient beta calculated by multivariable regression analysis adjusted for age, sex, BMI, HTN, MI, PAD, cerebro, and DM.

**Supplementary table 6. Unadjusted and adjusted associations between proteins in plasma-derived EV and other physical performance parameters.**

| **1) 5-times-sit-to-stand test** | | | | | | | | | | | | |
| --- | --- | --- | --- | --- | --- | --- | --- | --- | --- | --- | --- | --- |
| **at baseline** | | | | | | **over a 2-year period** | | | | | | |
| **Univariate** | | | **Multivariate** | | | **Univariate** | | | **Multivariate** | | | |
| **Protein** | **ꞵ** | ***p-*value** | **Protein** | **ꞵ** | ***p-*value** | **Protein** | **ꞵ** | ***p-*value** | **Protein** | **ꞵ** | | ***p-*value** |
| **HSPA5** | -0.280 | **0.008** | **PZP** | -0.317 | **0.011** | **ECM1** | -0.426 | **0.000** | **ECM1** | -0.407 | | **0.000** |
| **KNG1** | 0.254 | **0.017** | **MASP1** | -0.253 | **0.021** | **APOE** | -0.350 | **0.001** | **APOE** | -0.359 | | **0.001** |
| **MASP1** | -0.241 | **0.024** | **HSPA5** | -0.245 | **0.025** | **CLEC3B** | 0.344 | **0.001** | **CLEC3B** | 0.339 | | **0.002** |
| **LRP1** | -0.239 | **0.025** | **SERPINA6** | -0.228 | **0.039** | **TGFBI** | 0.324 | **0.002** | **MASP1** | 0.329 | | **0.002** |
| **PZP** | -0.220 | **0.039** | **AHSG** | 0.219 | **0.045** | **LBP** | 0.322 | **0.002** | **HRG** | 0.327 | | **0.003** |
| **GSN** | -0.217 | **0.042** |  |  |  | **HRG** | 0.317 | **0.003** | **APOC3** | -0.317 | | **0.003** |
| **HBD** | 0.214 | **0.046** |  |  |  | **MASP1** | 0.306 | **0.004** | **SERPINA4** | 0.299 | | **0.005** |
|  |  |  |  |  |  | **SERPINA4** | 0.297 | **0.005** | **LBP** | 0.303 | | **0.006** |
|  |  |  |  |  |  | **APOC3** | -0.295 | **0.005** | **TGFBI** | 0.299 | | **0.007** |
|  |  |  |  |  |  | **HP** | -0.267 | **0.012** | **HP** | -0.246 | | **0.023** |
|  |  |  |  |  |  | **GPLD1** | -0.258 | **0.015** | **GPLD1** | -0.251 | | **0.024** |
|  |  |  |  |  |  | **APOD** | -0.250 | **0.019** | **APOD** | -0.240 | | **0.025** |
|  |  |  |  |  |  | **PROS1** | 0.249 | **0.019** | **LRP1** | 0.241 | | **0.026** |
|  |  |  |  |  |  | **C1QC** | -0.246 | **0.021** | **PROS1** | 0.238 | | **0.027** |
|  |  |  |  |  |  | **ADIPOQ** | 0.242 | **0.023** | **C8B** | 0.232 | | **0.029** |
|  |  |  |  |  |  | **C8B** | 0.227 | **0.034** | **PMEL** | 0.235 | | **0.030** |
|  |  |  |  |  |  | **BNC2** | -0.220 | **0.039** | **C1QC** | -0.230 | | **0.036** |
|  |  |  |  |  |  | **LRP1** | 0.217 | **0.042** | **ADIPOQ** | 0.230 | | **0.039** |
|  |  |  |  |  |  |  |  |  | **BNC2** | -0.222 | | **0.041** |
|  |  |  |  |  |  |  |  |  | **CPB2** | 0.219 | | **0.042** |
|  |  |  |  |  |  |  |  |  | **APOA1** | 0.216 | | **0.047** |
| **2) SPPB** | | | | | | | | | | | | |
| **SPPB at baseline** | | | | | | **SPPB over a 2-year period** | | | | | | |
| **Univariate** | | | **Multivariate** | | | **Univariate** | | | **Multivariate** | | | |
| **Protein** | **ꞵ** | ***p-*value** | **Protein** | **ꞵ** | ***p-*value** | **Protein** | **ꞵ** | ***p-*value** | **Protein** | | **ꞵ** | ***p-*value** |
| **LRP1** | 0.282 | **0.009** | **MASP1** | 0.270 | **0.017** | **LBP** | -0.303 | **0.005** | **LBP** | | -0.305 | **0.006** |
| **F2** | 0.281 | **0.009** | **CRTAC1** | 0.261 | **0.017** | **C1QC** | 0.271 | **0.012** | **MASP1** | | -0.265 | **0.019** |
| **CRTAC1** | 0.276 | **0.011** | **SERPINA4** | 0.246 | **0.025** | **SERPINA4** | -0.248 | **0.022** | **SERPINA4** | | -0.253 | **0.021** |
| **SERPINA4** | 0.259 | **0.017** | **PLG** | 0.252 | **0.025** | **MASP1** | -0.229 | **0.035** | **C1QC** | | 0.256 | **0.023** |
| **HSPA5** | 0.251 | **0.021** | **HSPA5** | 0.243 | **0.029** |  |  |  | **HRG** | | -0.226 | **0.046** |
| **F13A1** | 0.245 | **0.024** | **LRP1** | 0.237 | **0.033** |  |  |  |  | |  |  |
| **PLG** | 0.229 | **0.035** | **F13A1** | 0.234 | **0.034** |  |  |  |  | |  |  |
| **MASP1** | 0.229 | **0.035** | **F2** | 0.230 | **0.047** |  |  |  |  | |  |  |

ꞵ; standardized coefficient beta calculated by multivariable regression analysis adjusted for age, sex, BMI, HTN, MI, PAD, cerebro, and DM.

**Supplementary table 13. Differences in EVs-related biomarker levels between stayed robust and non-to-sarcopenic groups over a 2-year period**

| **Based on changes in ASM/ht^2^**  **over a 2-year period** | **Protein** | **Stayed nonsarcopenic (n=11)** | **Non-to-sarcopenic (n=21)** | ***p-value*** |
| --- | --- | --- | --- | --- |
|  |  | **Normalized protein level (Mean ± SD)** | |  |
|  | HBD | 9.72 ± 0.67 | 8.71 ± 1.18 | 0.018 |
|  | C6 | 5.65 ± 0.36 | 6.05 ± 0.74 | 0.024 |
|  | CFI | 3.99 ± 0.35 | 4.59 ± 0.72 | 0.005 |
|  | CFH | 4.80 ± 0.41 | 5.26 ± 0.95 | 0.047 |
|  | HSPA5 | 2.78 ± 0.60 | 3.37 ± 0.86 | 0.009 |
|  | ECM1 | 5.93 ± 0.32 | 5.46 ± 0.64 | 0.041 |
|  | F12 | 4.73 ± 0.84 | 5.57 ± 0.63 | 0.007 |
|  | CRTAC1 | 2.73 ± 0.73 | 3.60 ± 0.84 | 0.015 |
|  | SERPINA10 | 3.14 ± 0.70 | 3.99 ± 0.76 | 0.004 |
|  | C7 | 6.70 ± 0.26 | 7.12 ± 0.62 | 0.025 |
|  | AMBP | 7.93 ± 0.30 | 8.27 ± 0.50 | 0.035 |
| **Based on changes in grip strength**  **over a 2-year period** | **Protein** | **Stayed nonsarcopenic (n=30)** | **Non-to-sarcopenic (n=28)** | ***p-value*** |
|  |  | **Normalized protein level (Mean ± SD)** | |  |
|  | THBS4 | 5.34 ± 0.73 | 4.78 ± 1.07 | 0.025 |
|  | HSPA5 | 3.01 ± 0.88 | 3.48 ± 0.99 | 0.041 |
|  | APOC1 | 3.06 ± 0.85 | 3.66 ± 0.93 | 0.016 |
|  | DBH | 3.13 ± 0.87 | 3.65 ± 0.93 | 0.027 |
|  | PRSS3 | 5.08 ± 1.24 | 5.58 ± 0.95 | 0.012 |
|  | F10 | 3.13 ± 0.72 | 3.62 ± 0.99 | 0.025 |
|  | APOE | 6.35 ± 0.67 | 6.81 ± 0.80 | 0.009 |
| **Based on changes in gait speed**  **over a 2-year period** | **Protein** | **Stayed nonsarcopenic (n=21)** | **Non-to-sarcopenic (n=10)** | ***p-value*** |
|  |  | **Normalized protein level (Mean ± SD)** | |  |
|  | SERPINA5 | 4.58 ± 0.34 | 5.16 ± 0.40 | 0.001 |
|  | CNDP1 | 2.86 ± 0.75 | 4.01 ± 0.96 | 0.002 |
|  | CFHR5 | 3.80 ± 0.48 | 4.36 ± 0.74 | 0.025 |
|  | SERPINA7 | 3.29 ± 1.04 | 3.96 ± 0.82 | 0.045 |
|  | C2 | 5.60 ± 0.24 | 6.05 ± 0.56 | 0.002 |
|  | HSPA5 | 2.95 ± 0.85 | 3.79 ± 0.86 | 0.011 |
|  | TGFBI | 4.51 ± 0.31 | 4.95 ± 0.76 | 0.020 |
|  | SELENOP | 4.55 ± 0.51 | 5.43 ± 0.86 | 0.002 |
|  | CPB2 | 3.43 ± 0.64 | 4.12 ± 0.65 | 0.007 |
|  | QSOX1 | 3.87 ± 0.52 | 4.53 ± 0.81 | 0.006 |
|  | ECM1 | 5.54 ± 0.71 | 6.13 ± 0.54 | 0.031 |
|  | F12 | 5.29 ± 0.46 | 5.88 ± 0.74 | 0.007 |
|  | CLEC3B | 4.05 ± 0.57 | 4.58 ± 0.54 | 0.029 |
|  | SERPIND1 | 5.90 ± 0.62 | 6.31 ± 0.31 | 0.018 |
|  | GPX3 | 3.25 ± 1.06 | 4.27 ± 0.75 | 0.005 |
|  | CRTAC1 | 2.91 ± 0.88 | 4.05 ± 1.27 | 0.042 |
|  | ADIPOQ | 4.71 ± 0.81 | 5.32 ± 0.86 | 0.040 |
|  | C4B | 8.70 ± 0.84 | 9.27 ± 0.71 | 0.034 |
|  | SERPINF1 | 6.22 ± 0.34 | 6.80 ± 0.44 | 0.002 |
|  | CPN1 | 6.06 ± 0.31 | 6.39 ± 0.38 | 0.016 |
|  | CP | 9.25 ± 0.24 | 9.54 ± 0.31 | 0.009 |
|  | AGT | 6.13 ± 0.39 | 6.46 ± 0.37 | 0.034 |
|  | C1R | 7.99 ± 0.40 | 8.22 ± 0.49 | 0.044 |
|  | C8A | 7.22 ± 0.41 | 7.64 ± 0.24 | 0.005 |
|  | C1S | 6.57 ± 0.34 | 6.83 ± 0.30 | 0.021 |
|  | F13A1 | 6.32 ± 0.41 | 6.71 ± 0.48 | 0.017 |
|  | AMBP | 8.23 ± 0.34 | 8.60 ± 0.45 | 0.025 |
|  | F2 | 8.09 ± 0.19 | 8.42 ± 0.21 | 0.000 |
|  | CFH | 7.75 ± 0.34 | 7.99 ± 0.33 | 0.031 |
|  | SERPING1 | 8.71 ± 0.38 | 8.99 ± 0.30 | 0.040 |
|  | GSN | 6.52 ± 0.44 | 7.05 ± 0.43 | 0.008 |
|  | CFB | 8.21 ± 0.22 | 8.45 ± 0.28 | 0.046 |
| **Based on changes in sarcopenia diagnosis over a 2-year period** | **Protein** | **Stayed nonsarcopenic (n=28)** | **Non-to-sarcopenic (n=24)** | ***p-value*** |
|  |  | **Normalized protein level (Mean ± SD)** | |  |
|  | HBD | 9.50 ± 0.79 | 8.88 ± 1.26 | 0.040 |
|  | LRP1 | 2.82 ± 0.90 | 3.64 ± 1.33 | 0.027 |
|  | CNDP1 | 3.00 ± 0.79 | 3.81 ± 1.49 | 0.006 |
|  | CFI | 4.15 ± 0.45 | 4.52 ± 0.75 | 0.033 |
|  | SERPINA7 | 3.26 ± 0.91 | 3.63 ± 0.92 | 0.025 |
|  | HSPA5 | 2.92 ± 0.81 | 3.58 ± 0.86 | 0.001 |
|  | SELENOP | 4.69 ± 0.63 | 5.03 ± 0.78 | 0.035 |
|  | MMRN1 | 3.43 ± 1.40 | 4.09 ± 1.10 | 0.043 |
|  | CPB2 | 3.38 ± 0.58 | 3.85 ± 0.89 | 0.038 |
|  | F12 | 5.10 ± 0.69 | 5.71 ± 0.70 | 0.003 |
|  | CLEC3B | 4.03 ± 0.63 | 4.39 ± 0.59 | 0.029 |
|  | GPX3 | 3.24 ± 0.94 | 3.86 ± 0.93 | 0.009 |
|  | CRTAC1 | 2.83 ± 0.84 | 3.80 ± 1.09 | 0.006 |
|  | SERPINA10 | 3.37 ± 0.67 | 4.09 ± 0.88 | 0.002 |
|  | C1QA | 7.22 ± 0.93 | 6.54 ± 1.18 | 0.047 |
|  | C4B | 8.69 ± 0.76 | 9.14 ± 0.63 | 0.002 |
|  | CPN1 | 6.09 ± 0.30 | 6.25 ± 0.44 | 0.045 |
|  | CP | 9.25 ± 0.25 | 9.43 ± 0.38 | 0.027 |
|  | C1S | 6.55 ± 0.30 | 6.69 ± 0.47 | 0.026 |
|  | C9 | 6.34 ± 0.52 | 6.61 ± 0.77 | 0.048 |
|  | F2 | 8.11 ± 0.26 | 8.27 ± 0.34 | 0.020 |

p-values were calculated using the Mann-Whitney test.
