# Supplementary material for: Plasma Extracellular Vesicles‐Derived Complement Proteins as Biomarkers of Sarcopenia Progression in Longitudinal Cohorts
Source: J Cachexia Sarcopenia Muscle. 2026 Jul 20;17(4):e70350. doi: 10.1002/jcsm.70350 (PMC13385205; doi:10.1002/jcsm.70350)
Supplement: Supplementary file 4 — TableS7: Ingenuity pathway analysis (IPA) of proteins from plasma derived EVs with ASM/ht2 at baseline. Table S8: Ingenuity pathway analysis (IPA) of proteins from plasma derived EVs with grip strength at baseline. Table S9: Ingenuity pathway analysis (IPA) of proteins from plasma derived EVs with gait speed at baseline. Table S10: Ingenuity pathway analysis (IPA) of proteins from plasma derived EVs with longitudinal changes in ASM. Table S11: Ingenuity pathway analysis (IPA) of proteins from plasma derived EVs with longitudinal changes in grip strength. Table S12: Ingenuity pathway analysis (IPA) of proteins from plasma derived EVs with longitudinal changes in gait speed. [file JCSM-17-e70350-s003.pdf]

Supplementary table 7. Ingenuity pathway analysis (IPA) of proteins from plasma derived EVs with ASM/ht2 at baseline

© 2000-2023 QIAGEN. All rights reserved.

Ingenuity Canonical Pathways	-log(p-value)	Ratio	z-score	Molecules
Growth Hormone Signaling	3.22	0.0282	#NUM!	A2M,IGFALS
Maturity Onset Diabetes of Young (MODY) Signaling	3.15	0.026	#NUM!	ADIPOQ,APOB
LXR/RXR Activation	2.74	0.0163	#NUM!	APOB,LBP
IL-6 Signaling	2.7	0.0155	#NUM!	A2M,LBP
Glucocorticoid Receptor Signaling	2.62	0.00554	#NUM!	A2M,KRT10,KRT14
Acute Phase Response Signaling	2.4	0.0109	#NUM!	A2M,LBP
Hepatic Fibrosis / Hepatic Stellate Cell Activation	2.37	0.0105	#NUM!	A2M,LBP
B Cell Development	2.24	0.00905	#NUM!	IGKV1-6,IGLV3-9
IL-15 Signaling	2.09	0.00755	#NUM!	IGKV1-6,IGLV3-9
FcγRIIB Signaling in B Lymphocytes	2.07	0.00735	#NUM!	IGKV1-6,IGLV3-9
p70S6K Signaling	1.94	0.00627	#NUM!	IGKV1-6,IGLV3-9
PI3K Signaling in B Lymphocytes	1.92	0.00615	#NUM!	IGKV1-6,IGLV3-9
B Cell Receptor Signaling	1.82	0.00543	#NUM!	IGKV1-6,IGLV3-9
Coagulation System	1.74	0.0286	#NUM!	A2M
Inhibition of Matrix Metalloproteases	1.7	0.0263	#NUM!	A2M
Systemic Lupus Erythematosus In B Cell Signaling Pathway	1.65	0.00443	#NUM!	IGKV1-6,IGLV3-9
iNOS Signaling	1.63	0.0222	#NUM!	LBP
Communication between Innate and Adaptive Immune Cells	1.63	0.00431	#NUM!	IGKV1-6,IGLV3-9
Altered T Cell and B Cell Signaling in Rheumatoid Arthritis	1.61	0.00424	#NUM!	IGKV1-6,IGLV3-9
Role of NFAT in Regulation of the Immune Response	1.46	0.00351	#NUM!	IGKV1-6,IGLV3-9
Systemic Lupus Erythematosus Signaling	1.43	0.00338	#NUM!	IGKV1-6,IGLV3-9
Role of JAK family kinases in IL-6-type Cytokine Signaling	1.4	0.013	#NUM!	LBP
Toll-like Receptor Signaling	1.4	0.013	#NUM!	LBP
Phospholipase C Signaling	1.36	0.00309	#NUM!	IGKV1-6,IGLV3-9
LPS-stimulated MAPK Signaling	1.36	0.0118	#NUM!	LBP
FXR/RXR Activation	1.2	0.008	#NUM!	APOB
Atherosclerosis Signaling	1.19	0.00781	#NUM!	APOB
Type II Diabetes Mellitus Signaling	1.12	0.00662	#NUM!	ADIPOQ
Germ Cell-Sertoli Cell Junction Signaling	1.08	0.00602	#NUM!	A2M
Macrophage Classical Activation Signaling Pathway	1.04	0.00556	#NUM!	LBP
PPARα/RXRα Activation	1.04	0.00546	#NUM!	ADIPOQ
Hepatic Cholestasis	1.03	0.00541	#NUM!	LBP
Production of Nitric Oxide and Reactive Oxygen Species in Macrophages	1.03	0.00532	#NUM!	APOB
Sertoli Cell-Sertoli Cell Junction Signaling	0.996	0.00495	#NUM!	A2M
Clathrin-mediated Endocytosis Signaling	0.987	0.00485	#NUM!	APOB
LPS/IL-1 Mediated Inhibition of RXR Function	0.959	0.0045	#NUM!	LBP
IL-12 Signaling and Production in Macrophages	0.943	0.00437	#NUM!	APOB
Osteoarthritis Pathway	0.939	0.00431	#NUM!	ADIPOQ
Actin Cytoskeleton Signaling	0.924	0.00417	#NUM!	LBP
AMPK Signaling	0.924	0.00415	#NUM!	ADIPOQ
Phagosome Formation	0.521	0.00149	#NUM!	LBP

Supplementary table 8. Ingenuity pathway analysis (IPA) of proteins from plasma derived EVs with grip strength at baseline

© 2000-2023 QIAGEN. All rights reserved.

Ingenuity Canonical Pathways	-log(p-value)	Ratio	z-score	Molecules
Complement System	8.29	0.111	#NUM!	C5,C8A,C8G,MASP1
Systemic Lupus Erythematosus Signaling	6.15	0.0102	#NUM!	C5,C8A,C8G,IGHG1,IGHV3-49,IGLC7
Multiple Sclerosis Signaling Pathway	5.19	0.019	-2	C5,C8A,C8G,MASP1
Role of Macrophages, Fibroblasts and Endothelial Cells in Rheumatoid Arthritis	4.48	0.0126	#NUM!	C5,IGHG1,IGLC7,PRSS3
B Cell Development	3.52	0.0136	#NUM!	IGHG1,IGHV3-49,IGLC7
Primary Immunodeficiency Signaling	3.35	0.0392	#NUM!	IGHG1,IGLC7
IL-15 Signaling	3.28	0.0113	#NUM!	IGHG1,IGHV3-49,IGLC7
FcγRIIB Signaling in B Lymphocytes	3.25	0.011	#NUM!	IGHG1,IGHV3-49,IGLC7
p70S6K Signaling	3.05	0.0094	#NUM!	IGHG1,IGHV3-49,IGLC7
PI3K Signaling in B Lymphocytes	3.03	0.00923	#NUM!	IGHG1,IGHV3-49,IGLC7
B Cell Receptor Signaling	2.87	0.00815	#NUM!	IGHG1,IGHV3-49,IGLC7
Neutrophil Extracellular Trap Signaling Pathway	2.78	0.00758	#NUM!	C5,IGHG1,IGLC7
NADH Repair	2.73	0.333	#NUM!	GAPDH
Neuroprotective Role of THOP1 in Alzheimer's Disease	2.67	0.0179	#NUM!	MASP1,PRSS3
Systemic Lupus Erythematosus In B Cell Signaling Pathway	2.62	0.00665	#NUM!	IGHG1,IGHV3-49,IGLC7
Communication between Innate and Adaptive Immune Cells	2.58	0.00647	#NUM!	IGHG1,IGHV3-49,IGLC7
Altered T Cell and B Cell Signaling in Rheumatoid Arthritis	2.56	0.00636	#NUM!	IGHG1,IGHV3-49,IGLC7
Role of NFAT in Regulation of the Immune Response	2.33	0.00526	#NUM!	IGHG1,IGHV3-49,IGLC7
Macrophage Alternative Activation Signaling Pathway	2.21	0.0104	#NUM!	IGHG1,IGLC7
Phospholipase C Signaling	2.18	0.00463	#NUM!	IGHG1,IGHV3-49,IGLC7
Hematopoiesis from Pluripotent Stem Cells	2.04	0.00847	#NUM!	IGHG1,IGLC7
Allograft Rejection Signaling	2	0.00803	#NUM!	IGHG1,IGLC7
Autoimmune Thyroid Disease Signaling	2	0.008	#NUM!	IGHG1,IGLC7
Glutaryl-CoA Degradation	1.93	0.0526	#NUM!	CA1
Glycolysis I	1.83	0.0417	#NUM!	GAPDH
Gluconeogenesis I	1.81	0.04	#NUM!	GAPDH
Tryptophan Degradation III (Eukaryotic)	1.8	0.0385	#NUM!	CA1
Dendritic Cell Maturation	1.64	0.00519	#NUM!	IGHG1,IGLC7
SPINK1 Pancreatic Cancer Pathway	1.47	0.0182	#NUM!	PRSS3
SPINK1 General Cancer Pathway	1.43	0.0164	#NUM!	PRSS3
IL-7 Signaling Pathway	1.35	0.0137	#NUM!	IGHG1
Maturity Onset Diabetes of Young (MODY) Signaling	1.33	0.013	#NUM!	GAPDH
Role of JAK family kinases in IL-6-type Cytokine Signaling	1.33	0.013	#NUM!	SERPINA10
Phagosome Formation	1.2	0.00298	#NUM!	IGHG1,IGLC7
Airway Pathology in Chronic Obstructive Pulmonary Disease	1.17	0.00885	#NUM!	C8G
S100 Family Signaling Pathway	1.12	0.00267	#NUM!	IGHG1,IGLC7
Iron homeostasis signaling pathway	1.1	0.00758	#NUM!	HBB
Role of Pattern Recognition Receptors in Recognition of Bacteria and Viruses	1.06	0.00685	#NUM!	C5
Erythropoietin Signaling Pathway	1	0.00595	#NUM!	HBB
Granulocyte Adhesion and Diapedesis	0.987	0.00575	#NUM!	C5
Acute Phase Response Signaling	0.967	0.00543	#NUM!	C5
Agranulocyte Adhesion and Diapedesis	0.947	0.00518	#NUM!	C5
CDX Gastrointestinal Cancer Signaling Pathway	0.943	0.00513	#NUM!	CA1
IL-12 Signaling and Production in Macrophages	0.876	0.00437	#NUM!	IGHG1
Osteoarthritis Pathway	0.87	0.00431	#NUM!	PRG4
Pathogen Induced Cytokine Storm Signaling Pathway	0.719	0.00294	#NUM!	C5

Supplementary table 9. Ingenuity pathway analysis (IPA) of proteins from plasma derived EVs with gait speed at baseline

© 2000-2023 QIAGEN. All rights reserved.

Ingenuity Canonical Pathways	-log(p-value)	Ratio	z-score	Molecules
Macrophage Alternative Activation Signaling Pathway	5.12	0.0155	#NUM!	IGHG2,IGHM,THBS1
B Cell Development	4.94	0.0136	#NUM!	IGHG2,IGHM,IGLV1-47
IL-12 Signaling and Production in Macrophages	4.9	0.0131	#NUM!	IGHG2,IGHM,THBS1
IL-15 Signaling	4.71	0.0113	#NUM!	IGHG2,IGHM,IGLV1-47
FcγRIIB Signaling in B Lymphocytes	4.67	0.011	#NUM!	IGHG2,IGHM,IGLV1-47
p70S6K Signaling	4.47	0.0094	#NUM!	IGHG2,IGHM,IGLV1-47
PI3K Signaling in B Lymphocytes	4.44	0.00923	#NUM!	IGHG2,IGHM,IGLV1-47
B Cell Receptor Signaling	4.28	0.00815	#NUM!	IGHG2,IGHM,IGLV1-47
Primary Immunodeficiency Signaling	4.24	0.0392	#NUM!	IGHG2,IGHM
Systemic Lupus Erythematosus In B Cell Signaling Pathway	4.02	0.00665	#NUM!	IGHG2,IGHM,IGLV1-47
Communication between Innate and Adaptive Immune Cells	3.98	0.00647	#NUM!	IGHG2,IGHM,IGLV1-47
Altered T Cell and B Cell Signaling in Rheumatoid Arthritis	3.96	0.00636	#NUM!	IGHG2,IGHM,IGLV1-47
Role of NFAT in Regulation of the Immune Response	3.72	0.00526	#NUM!	IGHG2,IGHM,IGLV1-47
Systemic Lupus Erythematosus Signaling	3.67	0.00508	#NUM!	IGHG2,IGHM,IGLV1-47
Phospholipase C Signaling	3.55	0.00463	#NUM!	IGHG2,IGHM,IGLV1-47
Hematopoiesis from Pluripotent Stem Cells	2.91	0.00847	#NUM!	IGHG2,IGHM
Allograft Rejection Signaling	2.86	0.00803	#NUM!	IGHG2,IGHM
Autoimmune Thyroid Disease Signaling	2.86	0.008	#NUM!	IGHG2,IGHM
Role of Macrophages, Fibroblasts and Endothelial Cells in Rheumatoid Arthritis	2.65	0.00629	#NUM!	IGHG2,IGHM
Dendritic Cell Maturation	2.49	0.00519	#NUM!	IGHG2,IGHM
Neutrophil Extracellular Trap Signaling Pathway	2.47	0.00505	#NUM!	IGHG2,IGHM
Inhibition of Angiogenesis by TSP1	2.12	0.0312	#NUM!	THBS1
Phagosome Formation	2.02	0.00298	#NUM!	IGHG2,IGHM
S100 Family Signaling Pathway	1.93	0.00267	#NUM!	IGHG2,IGHM
IL-7 Signaling Pathway	1.76	0.0137	#NUM!	IGHM
p53 Signaling	1.64	0.0102	#NUM!	THBS1
Bladder Cancer Signaling	1.57	0.00877	#NUM!	THBS1
Ribonucleotide Reductase Signaling Pathway	1.4	0.00592	#NUM!	THBS1
Pulmonary Healing Signaling Pathway	1.34	0.00508	#NUM!	THBS1
Synaptogenesis Signaling Pathway	1.15	0.00324	#NUM!	THBS1
Pulmonary Fibrosis Idiopathic Signaling Pathway	1.13	0.00311	#NUM!	THBS1

Supplementary table 10. Ingenuity pathway analysis (IPA) of proteins from plasma derived EVs with longitudinal changes in ASM

© 2000-2023 QIAGEN. All rights reserved.

Ingenuity Canonical Pathways	-log(p-value)	Ratio	z-score	Molecules
B Cell Development	4.94	0.0136	#NUM!	IGHV3-15,IGHV5-51,IGLV6-57
IL-15 Signaling	4.71	0.0113	#NUM!	IGHV3-15,IGHV5-51,IGLV6-57
FcγRIIB Signaling in B Lymphocytes	4.67	0.011	#NUM!	IGHV3-15,IGHV5-51,IGLV6-57
p70S6K Signaling	4.47	0.0094	#NUM!	IGHV3-15,IGHV5-51,IGLV6-57
PI3K Signaling in B Lymphocytes	4.44	0.00923	#NUM!	IGHV3-15,IGHV5-51,IGLV6-57
B Cell Receptor Signaling	4.28	0.00815	#NUM!	IGHV3-15,IGHV5-51,IGLV6-57
Systemic Lupus Erythematosus In B Cell Signaling Pathway	4.02	0.00665	#NUM!	IGHV3-15,IGHV5-51,IGLV6-57
Communication between Innate and Adaptive Immune Cells	3.98	0.00647	#NUM!	IGHV3-15,IGHV5-51,IGLV6-57
Altered T Cell and B Cell Signaling in Rheumatoid Arthritis	3.96	0.00636	#NUM!	IGHV3-15,IGHV5-51,IGLV6-57
Role of NFAT in Regulation of the Immune Response	3.72	0.00526	#NUM!	IGHV3-15,IGHV5-51,IGLV6-57
Systemic Lupus Erythematosus Signaling	3.67	0.00508	#NUM!	IGHV3-15,IGHV5-51,IGLV6-57
Phospholipase C Signaling	3.55	0.00463	#NUM!	IGHV3-15,IGHV5-51,IGLV6-57
Inhibition of Angiogenesis by TSP1	2.12	0.0312	#NUM!	THBS1
Coagulation System	2.08	0.0286	#NUM!	A2M
Inhibition of Matrix Metalloproteases	2.04	0.0263	#NUM!	A2M
Growth Hormone Signaling	1.77	0.0141	#NUM!	A2M
p53 Signaling	1.64	0.0102	#NUM!	THBS1
Bladder Cancer Signaling	1.57	0.00877	#NUM!	THBS1
IL-6 Signaling	1.52	0.00775	#NUM!	A2M
Germ Cell-Sertoli Cell Junction Signaling	1.41	0.00602	#NUM!	A2M
Ribonucleotide Reductase Signaling Pathway	1.4	0.00592	#NUM!	THBS1
Acute Phase Response Signaling	1.37	0.00543	#NUM!	A2M
Hepatic Fibrosis / Hepatic Stellate Cell Activation	1.35	0.00526	#NUM!	A2M
Macrophage Alternative Activation Signaling Pathway	1.34	0.00518	#NUM!	THBS1
Pulmonary Healing Signaling Pathway	1.34	0.00508	#NUM!	THBS1
Sertoli Cell-Sertoli Cell Junction Signaling	1.33	0.00495	#NUM!	A2M
IL-12 Signaling and Production in Macrophages	1.27	0.00437	#NUM!	THBS1
Synaptogenesis Signaling Pathway	1.15	0.00324	#NUM!	THBS1
Pulmonary Fibrosis Idiopathic Signaling Pathway	1.13	0.00311	#NUM!	THBS1
Glucocorticoid Receptor Signaling	0.91	0.00185	#NUM!	A2M

Supplementary table 11. Ingenuity pathway analysis (IPA) of proteins from plasma derived EVs with longitudinal changes in grip strength

© 2000-2023 QIAGEN. All rights reserved.

Ingenuity Canonical Pathways	-log(p-value)	Ratio	z-score	Molecules
B Cell Development	5.84	0.0181	#NUM!	IGHG1,IGHV3-7,IGHV3-74,IGHV6-1
Systemic Lupus Erythematosus Signaling	5.7	0.00846	#NUM!	IGHG1,IGHV3-7,IGHV3-74,IGHV6-1,PRPF19
IL-15 Signaling	5.53	0.0151	#NUM!	IGHG1,IGHV3-7,IGHV3-74,IGHV6-1
FcγRIIB Signaling in B Lymphocytes	5.48	0.0147	#NUM!	IGHG1,IGHV3-7,IGHV3-74,IGHV6-1
p70S6K Signaling	5.21	0.0125	#NUM!	IGHG1,IGHV3-7,IGHV3-74,IGHV6-1
PI3K Signaling in B Lymphocytes	5.17	0.0123	#NUM!	IGHG1,IGHV3-7,IGHV3-74,IGHV6-1
B Cell Receptor Signaling	4.96	0.0109	#NUM!	IGHG1,IGHV3-7,IGHV3-74,IGHV6-1
Systemic Lupus Erythematosus In B Cell Signaling Pathway	4.61	0.00887	#NUM!	IGHG1,IGHV3-7,IGHV3-74,IGHV6-1
Communication between Innate and Adaptive Immune Cells	4.56	0.00862	#NUM!	IGHG1,IGHV3-7,IGHV3-74,IGHV6-1
Altered T Cell and B Cell Signaling in Rheumatoid Arthritis	4.53	0.00847	#NUM!	IGHG1,IGHV3-7,IGHV3-74,IGHV6-1
Role of NFAT in Regulation of the Immune Response	4.21	0.00702	#NUM!	IGHG1,IGHV3-7,IGHV3-74,IGHV6-1
Phospholipase C Signaling	4	0.00617	#NUM!	IGHG1,IGHV3-7,IGHV3-74,IGHV6-1
Neutrophil Extracellular Trap Signaling Pathway	1.93	0.00505	#NUM!	C1QC,IGHG1
Complement System	1.82	0.0278	#NUM!	C1QC
Spliceosomal Cycle	1.68	0.0204	#NUM!	PRPF19
Primary Immunodeficiency Signaling	1.66	0.0196	#NUM!	IGHG1
IL-7 Signaling Pathway	1.51	0.0137	#NUM!	IGHG1
Role of Pattern Recognition Receptors in Recognition of Bacteria and Viruses	1.22	0.00685	#NUM!	C1QC
Acute Phase Response Signaling	1.12	0.00543	#NUM!	C1QC
Macrophage Alternative Activation Signaling Pathway	1.1	0.00518	#NUM!	IGHG1
Multiple Sclerosis Signaling Pathway	1.06	0.00476	#NUM!	C1QC
IL-12 Signaling and Production in Macrophages	1.03	0.00437	#NUM!	IGHG1
Hematopoiesis from Pluripotent Stem Cells	1.01	0.00424	#NUM!	IGHG1
Allograft Rejection Signaling	0.991	0.00402	#NUM!	IGHG1
Autoimmune Thyroid Disease Signaling	0.991	0.004	#NUM!	IGHG1
Role of Macrophages, Fibroblasts and Endothelial Cells in Rheumatoid Arthritis	0.893	0.00314	#NUM!	IGHG1
Dendritic Cell Maturation	0.812	0.0026	#NUM!	IGHG1
Phagosome Formation	0.595	0.00149	#NUM!	IGHG1
S100 Family Signaling Pathway	0.554	0.00134	#NUM!	IGHG1

Supplementary table 12. Ingenuity pathway analysis (IPA) of proteins from plasma derived EVs with longitudinal changes in gait speed

© 2000-2023 QIAGEN. All rights reserved.

Ingenuity Canonical Pathways	-log(p-value)	Ratio	z-score	Molecules
Acute Phase Response Signaling	12.7	0.0489	#NUM!	A2M,C1QA,C2,C4A/C4B,CP,F2,MBL2,SAA4,SERPINF1
p70S6K Signaling	10.6	0.0282	#NUM!	F2,IGHG1,IGHV1-18,IGHV3-30,IGHV3-43,IGHV3-74,IGHV5-51,IGKV3-20,IGLC7
B Cell Development	10.2	0.0362	#NUM!	IGHG1,IGHV1-18,IGHV3-30,IGHV3-43,IGHV3-74,IGHV5-51,IGKV3-20,IGLC7
IL-15 Signaling	9.61	0.0302	#NUM!	IGHG1,IGHV1-18,IGHV3-30,IGHV3-43,IGHV3-74,IGHV5-51,IGKV3-20,IGLC7
FcγRIIB Signaling in B Lymphocytes	9.52	0.0294	#NUM!	IGHG1,IGHV1-18,IGHV3-30,IGHV3-43,IGHV3-74,IGHV5-51,IGKV3-20,IGLC7
PI3K Signaling in B Lymphocytes	8.91	0.0246	#NUM!	IGHG1,IGHV1-18,IGHV3-30,IGHV3-43,IGHV3-74,IGHV5-51,IGKV3-20,IGLC7
B Cell Receptor Signaling	8.49	0.0217	#NUM!	IGHG1,IGHV1-18,IGHV3-30,IGHV3-43,IGHV3-74,IGHV5-51,IGKV3-20,IGLC7
Systemic Lupus Erythematosus In B Cell Signaling Pathway	7.8	0.0177	#NUM!	IGHG1,IGHV1-18,IGHV3-30,IGHV3-43,IGHV3-74,IGHV5-51,IGKV3-20,IGLC7
Communication between Innate and Adaptive Immune Cells	7.7	0.0172	#NUM!	IGHG1,IGHV1-18,IGHV3-30,IGHV3-43,IGHV3-74,IGHV5-51,IGKV3-20,IGLC7
Altered T Cell and B Cell Signaling in Rheumatoid Arthritis	7.65	0.0169	#NUM!	IGHG1,IGHV1-18,IGHV3-30,IGHV3-43,IGHV3-74,IGHV5-51,IGKV3-20,IGLC7
Complement System	7.2	0.111	-1	C1QA,C2,C4A/C4B,MBL2
Role of NFAT in Regulation of the Immune Response	7.01	0.014	#NUM!	IGHG1,IGHV1-18,IGHV3-30,IGHV3-43,IGHV3-74,IGHV5-51,IGKV3-20,IGLC7
Systemic Lupus Erythematosus Signaling	6.89	0.0135	#NUM!	IGHG1,IGHV1-18,IGHV3-30,IGHV3-43,IGHV3-74,IGHV5-51,IGKV3-20,IGLC7
Phospholipase C Signaling	6.59	0.0123	#NUM!	IGHG1,IGHV1-18,IGHV3-30,IGHV3-43,IGHV3-74,IGHV5-51,IGKV3-20,IGLC7
Coagulation System	5.13	0.0857	#NUM!	A2M,F13A1,F2
Extrinsic Prothrombin Activation Pathway	3.86	0.125	#NUM!	F13A1,F2
LXR/RXR Activation	3.49	0.0244	#NUM!	C4A/C4B,SAA4,SERPINF1
FXR/RXR Activation	3.47	0.024	#NUM!	C4A/C4B,SAA4,SERPINF1
Intrinsic Prothrombin Activation Pathway	3.04	0.0488	#NUM!	F13A1,F2
Primary Immunodeficiency Signaling	2.85	0.0392	#NUM!	IGHG1,IGLC7
Neutrophil Extracellular Trap Signaling Pathway	2.05	0.00758	#NUM!	C1QA,IGHG1,IGLC7
Role of Pattern Recognition Receptors in Recognition of Bacteria and Viruses	1.95	0.0137	#NUM!	C1QA,MBL2
Macrophage Alternative Activation Signaling Pathway	1.73	0.0104	#NUM!	IGHG1,IGLC7
Clathrin-mediated Endocytosis Signaling	1.67	0.00971	#NUM!	F2,SAA4
Multiple Sclerosis Signaling Pathway	1.66	0.00952	#NUM!	C1QA,C2
IL-12 Signaling and Production in Macrophages	1.59	0.00873	#NUM!	IGHG1,SAA4
Hematopoiesis from Pluripotent Stem Cells	1.56	0.00847	#NUM!	IGHG1,IGLC7
Allograft Rejection Signaling	1.52	0.00803	#NUM!	IGHG1,IGLC7
Autoimmune Thyroid Disease Signaling	1.52	0.008	#NUM!	IGHG1,IGLC7
Docosahexaenoic Acid (DHA) Signaling	1.39	0.0263	#NUM!	SERPINF1
Inhibition of Matrix Metalloproteases	1.39	0.0263	#NUM!	A2M
Role of Macrophages, Fibroblasts and Endothelial Cells in Rheumatoid Arthritis	1.33	0.00629	#NUM!	IGHG1,IGLC7
S100 Family Signaling Pathway	1.33	0.00401	#NUM!	IGHG1,IGLC7,SERPINF1
Dendritic Cell Maturation	1.18	0.00519	#NUM!	IGHG1,IGLC7
Growth Hormone Signaling	1.12	0.0141	#NUM!	A2M
IL-7 Signaling Pathway	1.11	0.0137	#NUM!	IGHG1
PEDF Signaling	1.05	0.0119	#NUM!	SERPINF1
Role of Tissue Factor in Cancer	0.924	0.0087	#NUM!	F2
Atherosclerosis Signaling	0.883	0.00781	#NUM!	SAA4
IL-6 Signaling	0.879	0.00775	#NUM!	A2M
Iron homeostasis signaling pathway	0.87	0.00758	#NUM!	CP
Gα12/13 Signaling	0.866	0.00752	#NUM!	F2
Germ Cell-Sertoli Cell Junction Signaling	0.777	0.00602	#NUM!	A2M
Phagosome Formation	0.777	0.00298	#NUM!	IGHG1,IGLC7
Production of Nitric Oxide and Reactive Oxygen Species in Macrophages	0.728	0.00532	#NUM!	SAA4
Hepatic Fibrosis / Hepatic Stellate Cell Activation	0.724	0.00526	#NUM!	A2M
Sertoli Cell-Sertoli Cell Junction Signaling	0.699	0.00495	#NUM!	A2M
Thrombin Signaling	0.668	0.00455	#NUM!	F2
Wound Healing Signaling Pathway	0.636	0.0042	#NUM!	F2
Actin Cytoskeleton Signaling	0.633	0.00417	#NUM!	F2
Senescence Pathway	0.558	0.0034	#NUM!	SAA4
Synaptogenesis Signaling Pathway	0.539	0.00324	#NUM!	COMP
Pulmonary Fibrosis Idiopathic Signaling Pathway	0.523	0.00311	#NUM!	F2
Glucocorticoid Receptor Signaling	0.344	0.00185	#NUM!	A2M
FAK Signaling	0.219	0.00121	#NUM!	ECM1
